# Chromosomal organization and evolutionary history of *Mariner* transposable elements in Scarabaeinae coleopterans

**DOI:** 10.1186/1755-8166-6-54

**Published:** 2013-11-29

**Authors:** Sarah G Oliveira, Diogo C Cabral-de-Mello, Rita C Moura, Cesar Martins

**Affiliations:** 1 Morphology Department, Biosciences Institute, UNESP - São Paulo State University, Botucatu, SP 18618-970, Brazil; 2 Biology Department, Biosciences Institute, UNESP - São Paulo State University, Rio Claro, SP 13506-900, Brazil; 3 Department of Biology, Biological Sciences Institute, UPE - Pernambuco State University, Recife, PE 50100-130, Brazil

**Keywords:** Chromosomal rearrangements, Evolution, Heterochromatin, Horizontal transfer, Repetitive DNA, Transposition

## Abstract

**Background:**

With the aim to increase the knowledge on the evolution of coleopteran genomes, we investigated through cytogenetics and nucleotide sequence analysis *Mariner* transposons in three Scarabaeinae species (*Coprophanaeus cyanescens*, *C. ensifer* and *Diabroctis mimas*).

**Results:**

The cytogenetic mapping revealed an accumulation of *Mariner* transposon in the pericentromeric repetitive regions characterized as rich in heterochromatin and *C*_*0*_*t*-1 DNA fraction (DNA enriched with high and moderately repeated sequences). Nucleotide sequence analysis of *Mariner* revealed the presence of two major groups of *Mariner* copies in the three investigated coleoptera species.

**Conclusions:**

The *Mariner* is accumulated in the centromeric area of the coleopteran chromosomes probably as a consequence of the absence of recombination in the heterochromatic regions. Our analysis detected high diversification of *Mariner* sequences during the evolutionary history of the group. Furthermore, comparisons between the coleopterans sequences with other insects and mammals, suggest that the horizontal transfer (HT) could have acted in the spreading of the *Mariner* in diverse non-related animal groups.

## Background

The repetitive DNAs represent a significant fraction of eukaryotic genomes and are primarily enriched in the heterochromatic regions, although some of them were observed in euchromatic regions [1-4]. Among the repeated DNAs, the transposable elements (TEs) are DNA sequences capable of changing their location in the genome, moving from one site to another, which seems to benefit only the elements and, for a long time, they have been considered as a “parasitic” and/or “selfish” elements. However, TEs represent an evolutionary force that provides the potential conditions for the emergence of new genes, modify gene expression, and adaptation to new environmental challenges [5-7]. In this way, TEs have a major role shaping and influencing the structure and function of the genomes [8].

Among several groups of TEs, *Mariner*-like elements (MLEs) are a superfamily of DNA transposons that consists in a single gene without introns flanked by two terminal inverted repeat (TIR) of about 30 bp, performing a total length of approximately 1.300 bp. Each terminal repeat is flanked by a TA dinucleotide, resulted from duplication of the target site duplication (TSD) [9,10]. The *Mariner* transposase gene encodes a protein of 330–360 amino acids, which recognizes the TIRs and cuts both strands at each end, being responsible for transposition by excising, exchanging, and fusing DNAs in a coordinated manner [11,12].

The *Mariner* superfamily is probably the most widespread and diverse group of TEs found in animals, persisting in the genomes through evolutionary time [13]. The MLE history started with their discovery in insects (being observed in several orders), and now their distribution has been reported in multiple invertebrate and vertebrate genomes [9,14-16]. The high similarity between sequences from distantly related organisms, the incongruence between TE and phylogeny, and the unequal distribution of some *Mariner* subfamilies among closely related taxa indicate that the horizontal transfer (HT) contributed to this widespread distribution [17-20].

The combination of molecular and cytogenetic analyses has established that several transposable elements are associated with chromosomal rearrangements such as deletions, duplications, inversions, the formation of acentric fragments and dicentric chromosomes, recombination and translocations of host genomes. In *Drosophila*, species in which there are a variety of studies, two kinds of elements, *P* and *hobo*, are especially prone to induce chromosome rearrangements. However, other transposons also appear to mediate chromosome rearrangements; these include the elements BEL, *HeT-A*, *Mariner*, roo*,* Tango and *TART*[21-23].

The cytogenetic mapping of repetitive DNAs has improved the knowledge of genome organization and chromosomal differentiation during the evolutionary history of the species. On the other hand, the genome organization of repetitive DNAs has been poorly investigated in Coleoptera, with only one study involving the cytogenetic mapping of TEs [24]. With the aim to contribute to the knowledge of coleopteran genomes evolution at molecular and chromosomal level, we investigated the repetitive DNA fraction of three Scarabaeinae species (*Coprophanaeus cyanescens*, *C. ensifer* and *Diabroctis mimas*), cytogenetically characterized by the presence of large blocks of heterochromatin [19,25]. The subfamily Scarabaeinae comprises a diverse and cosmopolitan group of Coleoptera that play an important role in the conservation of ecosystems as seed dispersers, pollinators, and recyclers of organic matter [26]. The overall chromosomal distribution of repetitive DNAs was investigated through the chromosomal hybridization of *C*_*0*_*t*-1 DNA fraction (DNA enriched with high and moderately repeated sequences), and the genomic features of *Mariner* TEs were addressed through nucleotide sequencing and chromosomal mapping. The knowledge of the repetitive portion of Scarabainae genomes brings the opportunity to advance in studies of genome organization, species evolution, and chromosome evolution in coleopterans.

## Results

### Cytogenetic mapping of *C*_*0*_*t*-1 DNA and *Mariner*

**Figure 1:**
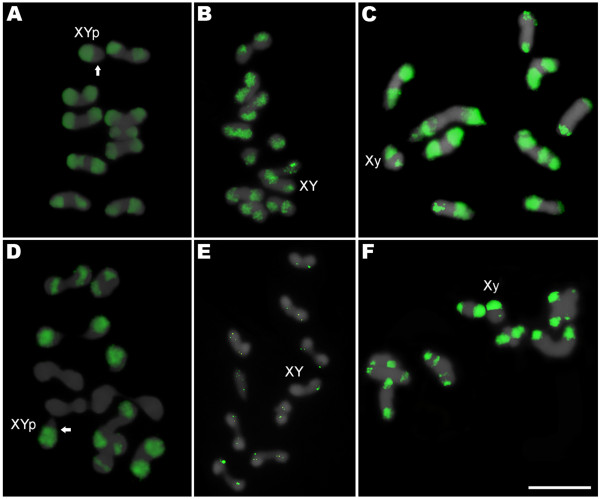
**Metaphases I of *Coprophanaeus *species submitted to FISH.** The metaphases of *C. cyanescens***(A,D)**, *C. ensifer***(B,E)** and *Diabroctis mimas***(C,F)** were probed with *C*_*0*_*t*-1 DNA **(A-C)** and *Mariner* transposable element **(D-F)**. The arrows indicate the chromosome Y **(A,D)**. Bar = 5 μm.

**Figure 2:**
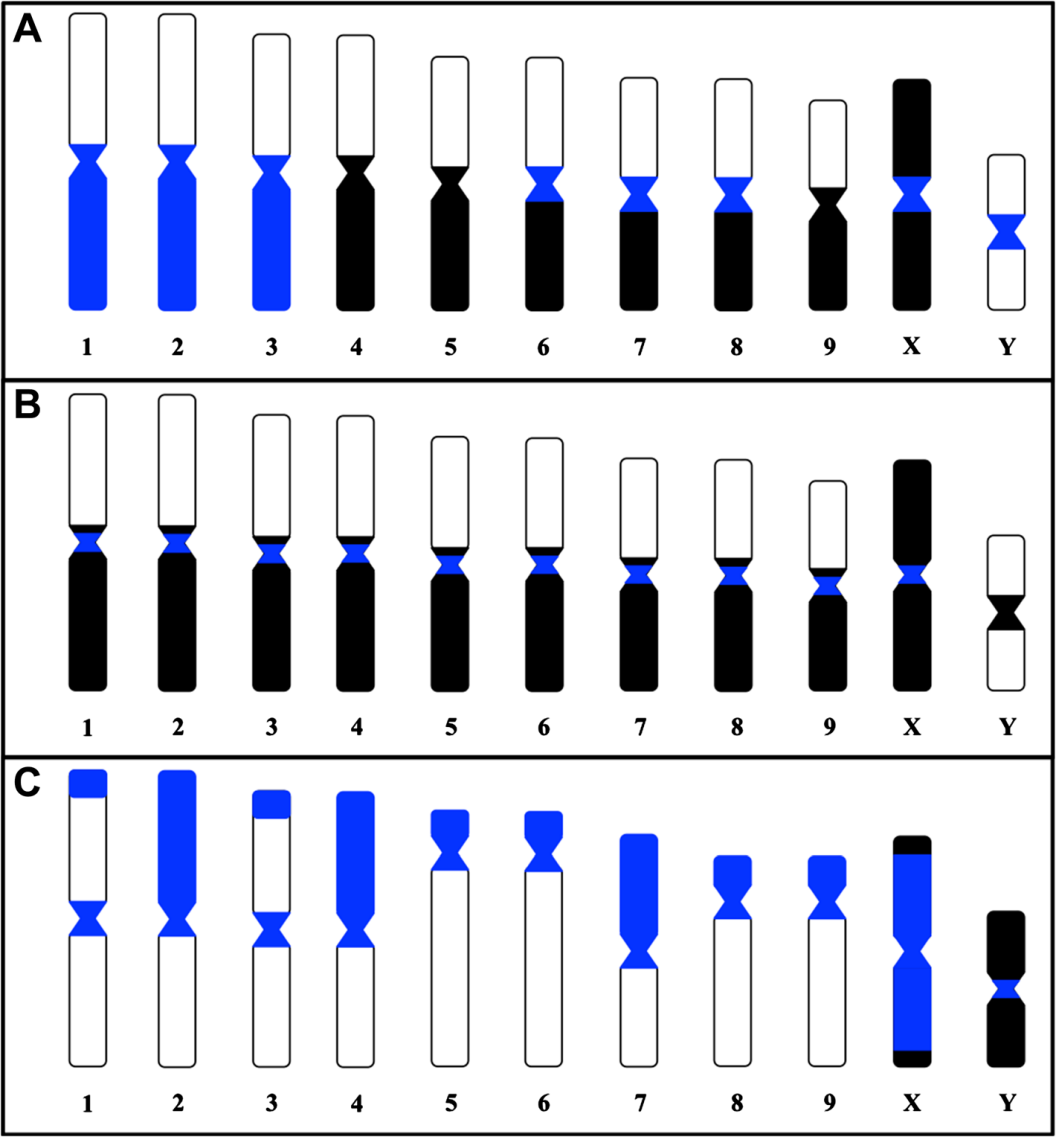
**Schematic ideogram showing the hybridization patterns described for *Coprophanaeus cyanescens *(A), *C. ensifer *(B) and *D. mimas *(C).** Black represents the distribution of heterochromatin revealed by C-banding according to previous works [25,27] and *C*_*0*_*t*-1 DNA. Blue represents the distribution of *Mariner* elements.

The *C*_*0*_*t*-1 DNA fraction was isolated from each genome of *C. cyanescens*, *C. ensifer* and *D. mimas*, and hybridized to their own chromosomes (Figure 1A-C). This *C*_*0*_*t*-1 DNA revealed positive hybridization in the long arms of all autosomes and X chromosome of the two *Coprophanaeus* species, and in the long arm of the Y chromosome of *C. ensifer* (Figure 1A, B). The *C*_*0*_*t*-1 DNA fraction hybridization in *D. mimas* evidenced large pericentromeric blocks in all autosomal pairs and in the sex chromosomes, extending to the short arms of some autosomal chromosomes (never to the long arm), including the terminal region, and in the terminal region of one autosomal pair (Figure 1C). Cross-species hybridization of *C*_*0*_*t*-1 DNA fraction showed positive hybridization only among species of the same genus, showing the same pattern observed for hybridization of probe originated from the same genome (as shown in Additional file 1: Figure S1). These patterns of *C*_*0*_*t*-1 DNA hybridization were similar to the data previously generated by C-banding on the three species [25,27]. However, it was not observed *C*_*0*_*t*-1 DNA hybridization in the C-positive banded centromeric region of the Y chromosome of *C. cyanescens*, as well as in the interstitial blocks of the short arms of three autosomal pairs and in the telomeric block in a small autosomal pair observed in *C. ensifer*[27]. Otherwise, *C*_*0*_*t*-1 DNA blocks observed in the terminal region of an autosomal pair and in the centromeric region of the Y chromosome of *D. mimas* were not observed by C-banding (Figure 2, [25]).

The FISH using probes of *Mariner* sequences in *C. cyanescens* labeled the X and Y sex chromosomes and four autosomal pairs with large pericentromeric blocks and three large autosomal pairs with pericentromeric labeling that also cover the long arm (Figure 1D). In *C. ensifer* the mapping of *Mariner* revealed small pericentromeric blocks in the X and in all autosomal chromosomes (Figure 1E). In *D. mimas* the pattern was similar to the obtained by *C*_*0*_*t*-1 DNA hybridization, however, the blocks observed were smaller (Figure 1F). A schematic ideogram showing the distribution of heterochromatic regions revealed by C-banding, and fluorescent *in situ* hybridization with *C*_*0*_*t*-1 DNA and *Mariner* sequences probes is presented in Figure 2.

### Analysis of *Mariner* sequences

**Figure 3:**
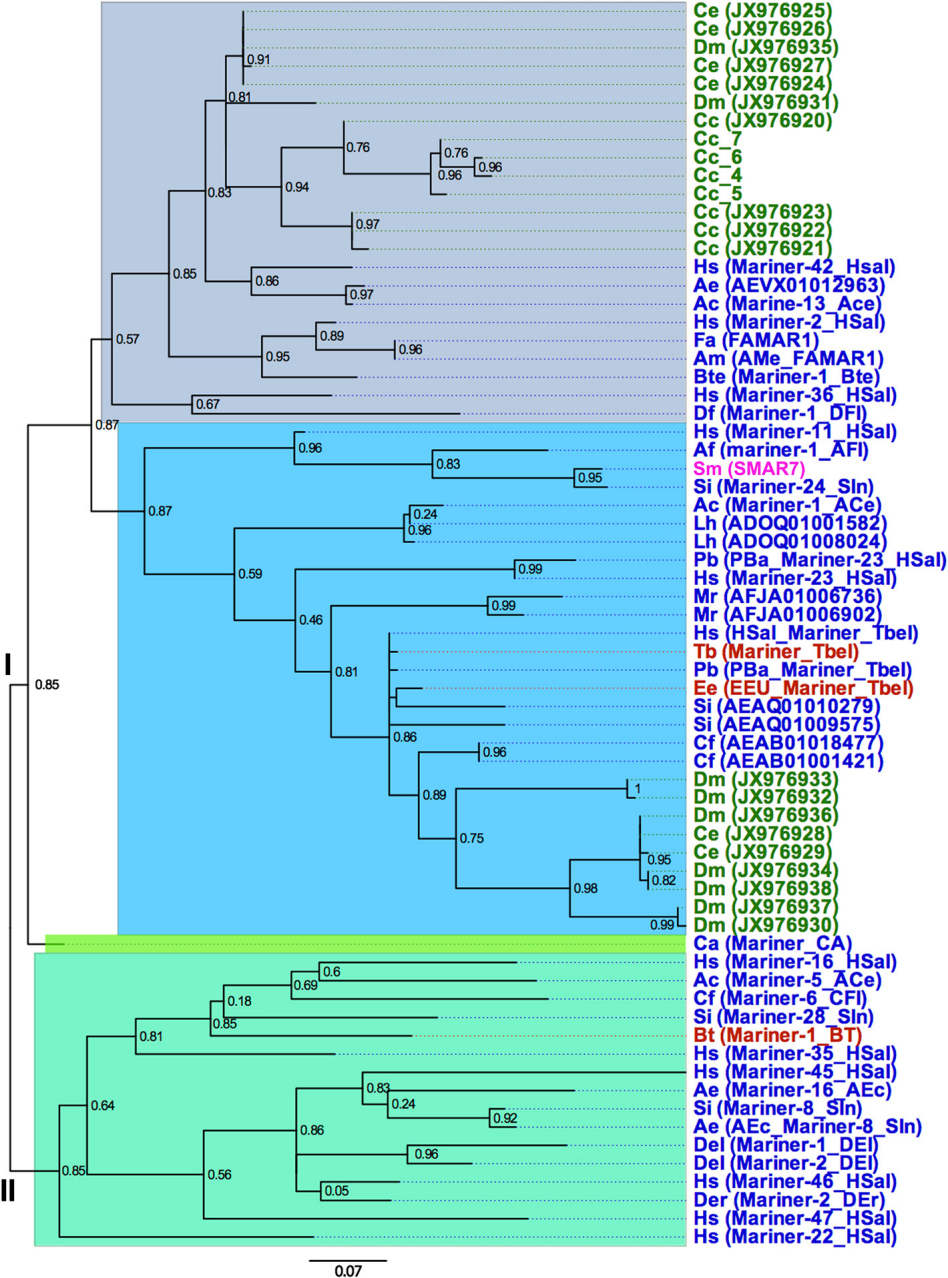
**Alignment guide tree of *Mariner *families.** The sequences obtained for beetles in this work are indicated in green. Except individual sequences with accession numbers provided, all other sequences are represented by consensus sequences deposited in Repbase, with their Repbase ID. The sequences are indicated in different colors considering if they are derived from mammals (red), planaria (pink), beetles (green) and other insects (blue). Species are Cc (*Coprophanaeus cyanesces*), Ce (*C. ensifer*), Dm (*Diabroctis mimas*), Ac (*Atta cephalotes*), Ae (*Acromyrmex echinatior*), Af (*Apis florea*), Am (*Apis mellifera*), Bte (*Bombus terrestris*), Bt (*Bos taurus*), Ca (*Chymomyza amoena*), Cf (*Camponotus floridanus*), Del (*Drosophila elegans*), Der (*Drosophila erecta*), Df (*Drosophila ficusphila*), Ee (*Erinaceus europeus*), Fa (*Forficula auricularia*), Hs (*Harpegnathos saltator*), Lh (*Linepithema humile*), Mr (*Megachile rotundata*), Pb (*Pogonomyrmex barbatus*), Si (*Solenopsis invicta*), Sm (*Schmidtea mediterranea*) and Tb (*Tupaia belangeri*). The sequences Cc_4 to Cc_7 are smaller than 200 bp and do not have GenBank accession numbers. The *Mariner* sequences are organized into two major groups (I and II, as indicated), and the groups are subdivided into branches (colored blocks). The branch support values are indicated on the nodes. The scale bar indicates the genetic distance.

Nucleotide transposase (partial DDE domain – region with three conserved aminoacid: Asparagine-Asparagine-Glutamine) sequences of approximately 230 bp were obtained for *C. cyanescens* (eight sequences), *C. ensifer* (six sequences) and *D. mimas* (nine sequences) (Additional file 2: Dataset S1). The comparative analysis of *Mariner* to several vertebrates and invertebrates showed that the *Mariner* sequences are organized into two major groups (I and II), and the group I is subdivided into 3 branches (Figure 3, Additional file 3: Figure S2). In the first branch, there is only the distribution of insect sequences, represented by flies (*Drosophila ficusphila*), ants (*Harpegnathos saltator*), bees (*Apis florea*, *Apis mellifera*), earwig (*Forficula auricularia*) and beetles (*C*. *cyanescens*, *C. ensifer*, *D. mimas*). In the second branch, it is observed the distribution of mammal sequences (*Erinaceus europeus*, *Tupaia belangeri*), planaria (*Schmidtea mediterranea*), and insects, represented by ants (*Atta cephalotes*, *Harpegnathos saltator*, *Linepithema humile*, *Solenopsis invicta*), bees (*Megachile rotundata*) and beetles (*C. ensifer*, *D. mimas*). In the third branch, it is observed only one sequence from the fly (*Chymomyza amoena*). The sequences of the group II are organized in one major branch and contain data of mammal (*Bos taurus*), and insects represented by flies (*Drosophila elegans*, *Drosophila erecta*) and ants (*Acromyrmex echinatior, Atta cephalotes*, *Camponotus floridanus*, *Harpegnathos saltator, Solenopsis invicta*).

In the first branch (group I sequences), the sequences of beetles showed similarity with other insect sequences, although they form a separated branch with high branch support value (0.87). In the second branch, the insect sequences are related to *Mariner1_Tbel* family from mammals. Even including the beetle sequences, it is clear the high similarity of sequences of the mammalians *Erinaceus europeus* and *Tupaia belangeri* with the genome of ants *Pogonomyrmex barbatus* and *Harpegnathos saltator* (higher than 92% compared with *Erinaceus europeus*, and higher than 98% compared with *Tupaia belangeri*) (Additional file 4: Dataset S2). The genetic distance within *Mariner1_Tbel* sequences between mammals and insects (including beetles) species were relatively low (0.017-0.359%) (Additional file 4: Dataset S2). In turn, the genetic distances observed within *Mariner-1_BT* sequences among insect species were 0.258-0.525% (Additional file 4: Dataset S2).

The *Mariner* sequences of beetles branched out in two into two groups (seen in the first and second branches of group I, Figure 3). Within the first branch, were obtained sequences from *C. cyanescens*, *C. ensifer* and *D. mimas*. However, within the second branch there were only obtained sequences from species *C. ensifer* and *D. mimas*. The genetic distance between this two beetle groups was relatively high (0.338-0.518).

## Discussion

### General aspects of heterochromatin and repeated DNAs organization

The presence of large blocks of heterochromatin and *C*_*0*_*t*-1 DNA fraction in the studied species suggests the occurrence of amplification of repetitive DNAs and/or heterochromatin transfer between the chromosomes during the karyotype differentiation of species as previously observed in other animals [28-31]. This statement is supported by the common pattern of heterochromatic blocks mainly located in pericentromeric areas in relates species, including coleopterans [32-34]. Most information concerning heterochromatin in coleopterans is focused on the description of chromosomal distribution with few data regarding its molecular content. The *C*_*0*_*t*-1 DNA fraction hybridization showed a general pattern coinciding with the data generated by C-banding [25,27], indicating that the heterochromatin is enriched in highly repetitive DNA. The presence of large blocks of *C*_*0*_*t*-1 suggests an abundance of repetitive sequences, and cross-species hybridization analysis among Phanaeini species evidences high conservation between the fractions of repetitive DNA within genera and divergence between the two different studied genera. However, the use of *C*_*0*_*t*-1 DNA fractions as probes in *Dichotomius* species (Coleoptera, Scarabaeidae) allowed the observation of heterochromatin distribution patterns highly conserved in the terminal/sub-terminal region and an extensive variation in relation to the pericentromeric heterochromatin [35]; which contrasts with the Phanaeini species studied. These data reinforce the intense evolutionary dynamics of the repeated DNA fraction by mutation, gene conversion, unequal crossing-over, circular replication and slippage replication [36-38] generating high divergence among taxa above the genus level.

### Chromosomal organization of *Mariner* transposable elements

It is a common observation that some transposable elements may be overabundant in specific regions of chromosomes, and the results obtained with the mapping of *Mariner* shows that these sequences are not randomly distributed and have accumulated in the heterochromatic areas. However, the accumulation of this element in euchromatic areas was recently reported in *Eyprepocnemis plorans*[4]. The accumulation of a large amount of copies in the heterochromatic regions can indicates a selection against insertions of TEs in euchromatin based on ectopic exchanges. Different major forces can affect TEs in heterochromatin and euchromatin regions of the genome, being that accumulation in heterochromatin regions explained by the absence of selection against insertional mutations in genetically inert regions, and stochastic accumulation of deleterious elements in regions with no recombination [1,2,39].

Possibly the absence of labeling in three autosomal pairs of *C. cyanescens* indicates that the evolutionary history of these sequences within the genome of the species follows a distinct pattern; possibly including suppression of recombination between these chromosomes with the other autosomes.

The accumulation of *Mariner* sequences in the pericentromeric regions is possibly due to the low rate of recombination characteristic of these regions, and could indicates that this element is enriched in regions where the damage of its insertion is reduced [22,40]. Although it is not possible to predict the possible role of these elements in Coleoptera they may be involved with the chromosomal rearrangements, as the occurrence of pericentromeric inversion observed in *D. mimas*. This species presents meta-submetacentric (pairs 1, 2, 3 and 7) and acrocentric (pairs 5, 6, 8 and 9) autosomal chromosomes [25], while *C. cyanescens* and *C. ensifer* have meta-submetacentric morphology for all autosomal chromosomes [27]. In *D. mimas* the presence of four acrocentric autosomal pairs indicates the occurrence of pericentromeric inversions unlike the standard meta-submetacentric karyotype described for the family Scarabaeidae [41]. Chromosomal rearrangements, as that observed in *D. mimas*, are possible a consequence of transposable elements, that were reported to be involved with various types of rearrangements by transposition and recombination [42-44].

Another approach to the accumulation of transposable elements is that the *Mariner* transposon could have been maintained in the pericentromeric region by presenting any functional role in the maintenance of this region [45]. For example, during the evolution of the genome, heterochromatic transposable elements may lose the ability to transpose and accumulate mutations and structural rearrangements, acquiring new functions [46,47]. Feschotte [6] proposed that the movement and accumulation of TEs, as well their derived proteins, have played an important role in the evolution of the genome. The association of TEs and the structure and/or function of centromeres seems to be an usual occurrence, and have been observed in diverse species [47,48].

The mapping of *Mariner* in the sex chromosomes of the three species could be related to the common spreading of the TEs in most heterochromatic areas of the genome, or the sex chromosomes can act as a refuge for transposable elements as previously reported [49-51]. Several genetic processes can cause an accumulation of TEs in genomic regions where crossing over is reduced or absent [36]. In some cases, for example, the sex chromosomes show the tendency of non-recombining in the genomic regions to accumulate transposable elements [52,53]. Another possibility is that, the recombination suppression itself could inhibit recombination in nearby regions of the sex chromosomes [53].

The transposition/selection model establishes that the distribution and abundance of TEs are indicative of their evolutionary history [36,54]. This process involves three stages: (i) invasion of the host genome, (ii) rapid spread by replicative transposition, and (iii) vertical inactivation and accumulation in the heterochromatin. Considering that hypothesis, the *Mariner* present in *C. cyanescens*, *C. ensifer* and *D. mimas* could be considered ancient because active and recently acquired elements are expected to be preferentially located in euchromatin. TEs are expected to be overabundant in the heterochromatin where recombination is strongly reduced, and because the TEs cannot be easily removed from heterochromatin once they have been inserted [55,56].

Besides the accumulation in the heterochromatin, the *Mariner* sequences hybridization patterns are quite different between the three species. This suggests that the chromosomes do not share a general pool of *Mariner* sequences, and could indicate a different evolutionary path after the emergence within each species.

### *Mariner* transposable elements in Scarabaeinae coleopterans

The *Mariner* sequences of Scarabaeinae coleopterans branched out into two groups, showing a relatively high genetic distance between them, indicating an early divergence from an ancestral element. This is consistent with previous studies, proposing that members of the *Tc1*/*Mariner* are probably monophyletic in origin, and diversified in various groups by accumulation of modifications and/or horizontal transfer mechanisms [9,57,58].

Probably, each TE copy of beetles has evolved independently of each other, according to the pattern of molecular evolution related for *Mariner* transposon. When divergent elements do exist, they display, as observed, a low percentage of similarity to the full-length sequences. This suggests that TEs are highly active within the genome, and that the highly divergent copies reflect relics of ancient mobilizations, as described to *Drosophila melanogaster*[53].

### *Mariner* horizontal transfer

*Mariner* transposable elements have been described in many arthropods, possibly spread by HT [17,20,59]. In general, the phylogenies based on *Mariner* sequences are not always congruent with the phylogenies of the taxa, suggesting the occurrence of HT [14,60].

The high sequence similarity between sequences from distantly related organisms, the incongruence between TE distribution and phylogeny, and the unequal distribution of some *Mariner* subfamilies among closely related taxa indicate that the HT contributed to this widespread distribution [18,20,61]. Several TEs have been introduced into mammal lineages through HT [62-65], including *Mariner*[19,66,67]). Comparative analyses of mammalian genomes show the presence of high amount of TEs, but their content could vary among the different lineages [18,68]. The genetic distance within *Mariner1_Tbel* sequences between mammals and insects species were relatively low, consistent with the phylogenetic distances between them and reinforces the occurrence of HT in the spread of these elements to different taxa [19].

Considering the *Mariner* tree topology clearly indicates the involvement of HT during the evolutionary history of insects and mammals, although it is not possible to show in which evolutionary moment this transfer occurred. Multiple mechanisms may be related to the spread of TE by horizontal transfer, using different types of vectors (external parasites, infectious agents, intracellular parasites and symbionts, DNA viruses, RNA viruses, retroviruses) [13,69,70]. Thus, for each described case of a proposed HT, could be implemented a model of transfer. Our results are consistent with the criteria of HT, and reveal interesting patterns of patchy distribution among animals, suggesting a repeated invasion of *Mariner* from insects to mammals.

## Conclusions

The relatively high genetic distances observed between the two classes of *Mariner* sequences of beetles, and their distribution in other animals indicate that these two classes had a early origin in the base of insect diversification, or considering the highest similarity for each group with other insect sequences (particularly ants), and also similarity to mammals in one of the groups, there is an evidence that the sequences may have originated by horizontal transfer.

## Methods

### Animals and DNA samples

Adult male samples of *Coprophanaeus cyanescens*, *C. ensifer* and *Diabroctis mimas* were collected in Caruaru, Igarassu, Paudalho and Saloá, Pernambuco State, Brazil. The animals were collected in the wild according to Brazilian laws for environmental protection (wild collection permit, MMA/IBAMA/SISBIO n. 2376–1). The experimental research on animals was approved by the ethics committee of Sao Paulo State University (Protocol no. 35/08 – CEEA/IBB/UNESP). The testes were fixed in Carnoy solution (3:1 ethanol: acetic acid) and then stored in freezer at −20°C. The DNA samples were obtained from living specimens immediately frozen in the freezer at −20°C. The procedure for extraction of genomic DNA followed, with minor modifications, the protocol described by Sambrook and Russel [71]. The quality and quantity of purified DNA were analyzed under electrophoresis and spectrophotometry.

### *C*_*0*_*t*-1 DNA preparation

*C*_*0*_*t*-1 DNA fractions were obtained from *C. cyanescens*, *C. ensifer* and *D. mimas* based on reassociation kinetics proposed by Zwick et al. [72] and the modifications described by Ferreira and Martins [73]. The *C*_*0*_*t*-1 DNA fractions obtained were labeled and used directly as probes for chromosome hybridization, being performed hybridizations within the same species and between different species.

### Isolation and characterization of *Mariner* TEs

The *Mariner* transposable elements were isolated thought polymerase chain reaction (PCR) with the set of primers MAR-188 F (5′ ATC TGR AGC TAT AAA TCA CT) and MAR-251R (5′ CAA AGA TGT CCT TGG GTG TG), designed based on conserved regions of the amino acid sequence of the putative transposase gene of the *Mariner* element [74]. The PCR products were cloned using pGEM-T kit (Promega, Madison, WI, USA) according to manufacturer’s recommendations. The recombinant plasmids were submitted to nucleotide sequencing using a sequencer model 3500 Genetic Analyzer (Applied Biosystems, Foster City, CA, USA).

The DNA sequences obtained were used as an initial query to searches against a database of repetitive DNA elements (Repbase database) (http://www.girinst.org/repbase/), which contains repetitive DNA sequences of various eukaryotic species [75]. Additionally, the obtained sequences were analyzed against the nucleotide collection of The National Center for Biotechnology (NCBI) (http://www.ncbi.nlm.nih.gov) using the Blast search tool. Family consensus sequences were constructed whenever possible. The analysis of DNA sequences were performed with web site LIRMM (Laboratoire Le d’Informatique, Robotique et de Microélectronique of Montpellier), available online at http://www.phylogeny.fr/[68,76,77]. The multiple alignments were performed using MUSCLE, while the alignment curation used Gblocks program to eliminate poorly aligned positions and divergent regions. Phylogenetic trees were built with neighbor joining (NJ) and confirmed as consistent with trees built by PhyML [78]. The *Mariner* protein coding sequences were searched against the Pfam database (http://pfam.sanger.ac.uk). For comparison, was also prepared a phylogenetics analysis using the Maximum Likehood, measuring the consistency by bootstrap using the program MEGA5 – Molecular Evolutionary Genetics Analysis (http://www.megasoftware.net/).

### Chromosome preparation and Fluorescence *in situ* hybridization (FISH)

Meiotic chromosomes for FISH were obtained from testes of *Coprophanaeus cyanescens*, *C. ensifer* and *Diabroctis mimas* according to the classic technique of squashing of testicular follicles using a drop of 45% acetic acid, and then dipped in liquid nitrogen to remove the coverslip.

The PCR products containing a pool of *Mariner* sequences, and the *C*_*0*_*t*-1 DNA fraction were labeled with biotin-11-dATP by nick translation using the Bionick Labeling System kit (Invitrogen, San Diego, CA, USA). The FISH protocol followed the adaptations described by Cabral-de-Mello et al. [79]. The probes were labeled with biotin-14-dATP and detected by avidin-FITC (fluorescein isothiocynate) conjugated (Sigma-Aldrich, St. Louis, MO, USA). The chromosomes were counterstained with 4,6-diamidino-2-phenylindole (DAPI) and the slides mounted with Vectashield (Vector, Burlingame, CA, USA). The images were captured using Olympus DP71 digital camera coupled to a BX61 Olympus microscope and DP Control program, and processed through Corel Photo-Paint 12 and Adobe Photoshop CS2.

## Competing interests

The authors declare that they have no competing interests.

## Authors’ contributions

SGO carried out the cytogenetic and molecular analysis and draft the manuscript. DCC helped in obtained cytogenetic data. RCM, CM and DCC conceived of the work, and participated in its design, drafted and revised the manuscript. All authors analyzed all results and read and approved the final manuscript.

## Supplementary Material

Additional file 1: Figure S1 Cross-species hybridization of *C*_*0*_*t*-1 DNA fraction in metaphases I of *Coprophanaeus* species. Probe of *C. ensifer* hybridized in *C. cyanescens* (a) and probe of *Coprophanaeus cyanescens* hybridized in *C. ensifer* (b). Bar = 5 μm. Click here for file

Additional file 2: Dataset S1 Sequence alignment of related *Mariner* families of diverse organisms retrieved from public databases and sequences obtained in the present work. The abbreviations correspond to the species names and IDs, as shown in the caption of Figure 3. Dashes represent indels. Click here for file

Additional file 3: Figure S2 Alignment guide tree of *Mariner* families based on Maximum Likehood. The taxa are the same as described in Figure 3. The bootstrap support values are indicated on the nodes. The scale bar indicates the genetic distance. Click here for file

Additional file 4: Dataset S2 Alignments pairwise similarity matrix of related *Mariner* families. The abbreviations correspond to the species names and IDs, as shown in Figure 3. Click here for file
