# Supplementary material for: Chromosomal organization and evolutionary history of Mariner transposable elements in Scarabaeinae coleopterans
Source: Mol Cytogenet. 2013 Nov 29;6:54. doi: 10.1186/1755-8166-6-54 (PMC3906913; doi:10.1186/1755-8166-6-54)

Additional File 1: Figure S1 - Cross-species hybridization of the C_0t-1 DNA fraction in metaphase I of *Coprophanaeus* species. Probe of *C. ensifer* hybridized to *C. cyanescens* (a) and probe of *C. cyanescens* hybridized to *C. ensifer* (b). The sex chromosome bivalents are indicated. Bar = 5µm.

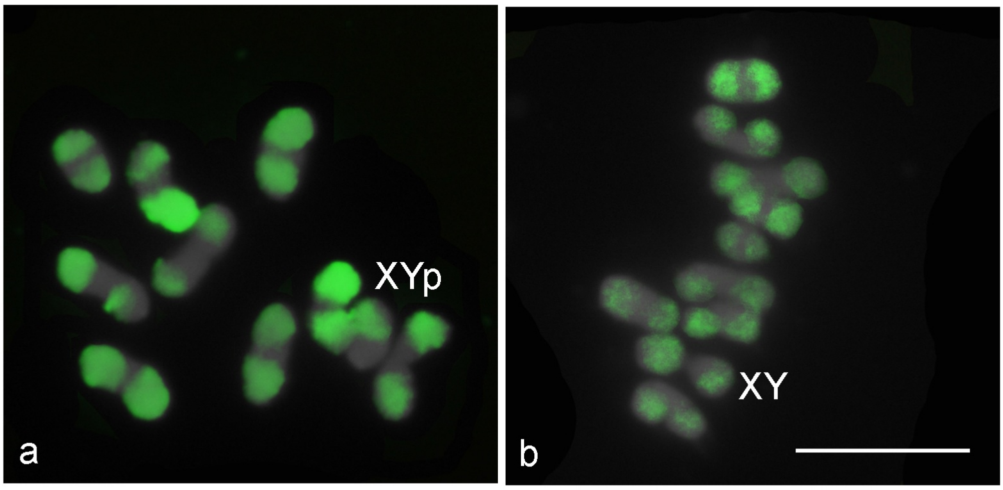

Supplement: Additional file 1: Figure S1 — Cross-species hybridization of C0t-1 DNA fraction in metaphases I of Coprophanaeus species. Probe of C. ensifer hybridized in C. cyanescens (a) and probe of Coprophanaeus cyanescens hybridized in C. ensifer (b). Bar = 5 μm. [file 1755-8166-6-54-S1.pdf]
