# Supplementary material for: Chromosomal organization and evolutionary history of Mariner transposable elements in Scarabaeinae coleopterans
Source: Mol Cytogenet. 2013 Nov 29;6:54. doi: 10.1186/1755-8166-6-54 (PMC3906913; doi:10.1186/1755-8166-6-54)
Supplement: Additional file 2: Dataset S1 — Sequence alignment of related Mariner families of diverse organisms retrieved from public databases and sequences obtained in the present work. The abbreviations correspond to the species names and IDs, as shown in the caption of Figure 3. Dashes represent indels. [file 1755-8166-6-54-S2.pdf]

Additional File 2: Dataset S1 - Sequence alignment of related *Mariner* families of diverse organisms retrieved from public databases and sequences obtained in the present work. The abbreviations correspond to species names and IDs as described in the caption of Figure 2. Dashes represent indels.

```
Hs (Mariner-24_SIn)         AAACAACCTCGAAGGCGGGATTGCATCCGAAGAAAATAAT------GCTCTCCGTATGGT
Hs (Mariner-45_HSal)        AGTCTGTGGCAAAGCCCACACTGACGCCCAGGAAGGTTAT------GCTGTGTGTTTGGT
Cf (Mariner-6_CFl)          TAACCACTTCGAAGGCAGGCTTACATCCGAAAAAGGTAAT------GCTCTGCATTTGGT
Hs (Mariner -35_HSal)       AAACCACACCGAAGCAAAACATCCATACGAAAAAAGTCAT------GCTGTGTATTTGGT
Df (Mariner-1-DF)           AAGCCACATCAAAGGCCGATATCCATCAGAGGAAAGTTAT------GCTGTCTGTTTGGT
Ac (Mariner-1_ACe)          AAAGGCAAGCAAAGGCGGAGATCCACCAAAAGAAGGTAAT------GCTGTCAATGTGGT
Lh (ADOQ01008024)           AAAGGCAAGCAAAGGCGGACATCCACCAAAAGAAGGTGAT------GCTGTCAATCTGGT
Lh (ADOQ01001582)           ----CACCGGAAAGGCGGACATCCACCAAAAGAAGGTGAT------GCTGTCAATCTGGT
Dm (JX976930)               ------TATCAAAAGCTGGTATTCATCATAGGAAGTTTAT------GCTCTCAGTGTGGT
Dm (JX976937)               ------TATCAAAAGCTGATATTCATCATAGGAAGTTTAT------GCTCTCAGTGTGGT
Dm (JX976938)               -----------AAAGCTGATATTCATCATAGAAAGTTTAA------GCTCTCACTGTGGT
Ce (JX976929)               -----------AAAGCTGGTATTCATCATAGAAAGTTTAT------GCTCTCACTGTGGT
Dm (jx976934)               ------TATCAAAAGCTGGTATTCATCATAGAAAGTTTAT------GCTCTCACTGTGGT
Ce (JX976928)               ------TATCAAAAGCTGATATTCATCATAGTAAGTTTAT------GCTCTCACTGTGGT
Dm (JX976936)               -----------AAAGCTGATATTCATCATAGAAAGTTTAT------GCTCTCACTGTGGT
Mr (AFJA01006902)           AAACGAGATCGAAACCAGAGATCCACCAGAGGTAGATTAT------GCTCTCTGTGTGGT
Mr (AFJA01006736)           AAACGAGATCGAAACCAGAGATCCACCACAGGAAGGTAAT------GCTCTCTGTGTGGT
Dm (JX976932)               -----------AAAGCTGGTATTCATCAGAAGAAGGTTAT------GCTCACAGTGTAGT
Dm (JX976933)               ------TATCAAAAGCTGGTATTCATCAGAAGAAGGTTAT------GCTCACAGTGTAGT
Si (AEAQ01010279)           ---CGACATCAAAAGCAAACATTCATCAGAGAAAAATTATGTTGTGGCTCTCAGTGTGGT
Si (AEAQ01009575)           ---CGACATCAAAAGCAAACATTCA---GAGGAAAGTTAT------GCTCTTAATGTGAT
Cf (AEAB01001421)           ---CAACTTCAAAAGCAGAACTTCATCAGAGGAAGATTAT------GCTCTCAGTGTGGT
Cf (AEAB01018477)           ---CAACTTCAAAAGCAGAACTTCATCAGAGGAAGATTAT------GCTCTCAGTGTGGT
Ee (EEu_Mariner_Tbel)       AAATGACATCGAAAGCAGACATTCATCAGAGGAAGGTTAT------GCTCTCAGTGTGGT
Pb (PBa_Mariner_Tbel)       AAACGACATCAAAAGCAAACATTCATCAGAGGAAGGTTAT------GCTCTCAGTGTGGT
Tb (Mariner_Tbel)           AAACGACATCGAAAGCAGACATTCATCAGAGGAAGGTTAT------GCTCTCAGTGTGGT
Hs (HSal_Mariner_Tbel)      AAACGACATCGAAAGCAGACATTCATCAGAGGAAGGTTAT------GCTCTCAGTGTGGT
Hs (Mariner-22_HSal)        AAACCACTTCGAAGGCAGGGCTTCACCCGAGCAAGATCAT------GCTCTCAATTTGGT
Ae (Mariner-16_AEc)         AAGCAATCGCAAAACCTGGATTGACGACGAAGAAGGTGAT------GTTGTGTGTGTGGT
Ae (AEc_Mariner-8_Sln)      AAACGACTTCAAAGCCTGGATTGATGGCAAATAAGGTGAT------GCTGTGTGTATGGT
Si (Mariner-8_SIn)          AAACGACTTCAAAGCCTGATTTGACGACAAATAAGGTGAT------GCTGTGTGTATGGT
Bte (Mariner-1_Bte)         AGACGACACCAAAAGCTGAGATTCACCA-AAAAAGATTAT------GCTGTCAGTTTGGT
Am (AMe_FAMAR1)             AAACAACATCAAAAGCTGGTATTCATCAAAAGAAGGTTTT------GTTATCAGTTTGGT
Fa (FAMAR1)                 AAACAACATCAAAAGCTGGTATTCATCAAAAGAAGGTTTT------GTTATCAGTTTGGT
Hs (Mariner-2_HSal)         AAACAACATCCAAAGCTGATATTCATCAAAAGAAAGTTAT------GTTATCAGTTTGGT
Hs (Mariner-42_HSal)        AAACCGTATCGAAAGCTGAATTGCATCAAAAAAAGATCAT------GCTGTCAATTTGGT
Ac (Mariner-13_ACe)         AAACCTCATCGAAAGCCGAACTGCATCAAAAAAAGATAAT------GCTGTCAATTTGGT
Ae (AEVX01012963)           AAACCTCATCGAAAGCTGAACTGCATCAAAAAAAGATAAT------GCTGTCAATTTGGT
Cc_4                        ----------AAAAGCTGGTATTCATC--AAAAAGATCAT------GTTGTCAATTTGGT
Cc_6                        ----TATCTCAAAAGCTGATATTCATCA-AAAAAGATCAT------GTTGTCAATTTGGT
Cc_5                        ----------AAAAGCTGGTATTCATCA-AAAAAGATCAT------GTTGTCAATTTGGT
Cc_7                        ----------AAAAGCTGGTATTCATCA-AAAAAGATCAT------GTTGTCAATTTGGT
Cc (JX976920)               ---TATCATCAAAAGCTGATATTCATCA-AAAAGGATCAT------GCTATCAGTTTGGT
Cc (JX976921)               ----------AAAAGCTGGTATTCATCA-AAAAGGATCAT------GCTATCAGTTTGGT
Cc (JX976922)               ---TATCATCAAAAGCTGGTATTCATCA-AAAAGGATCAT------GCTATCAGTTTGGT
Cc (JX976923)               ----------AAAAGCTGGTATTCATCA-AAAAGGATCAT------GCTATCAGTTTGGT
Dm (JX976931)               ----------AAAAGCTGGTATTCATCATA--AAGGTCAT------GCTCTCAATTTGGT
Ce (JX976927)               AAA--------GAAGCTGATATTCATCA-AAAAAGATCAT------GCTGTCAATTTGGT
Dm (JX976935)               ---TATCAAAAAAAGCTGATATTCATCA-AAAAAGATCAT------GCTGTCAATTTGGT
Ce (JX976926)               ----------AAAAGCTGATATTCATCA-AAAAAGATCAT------GCTGTCAATTTGGT
Ce (JX976924)               AAA-------AAAAGCTGATATTCATCA-AAAAAGGTCAT------GCTGTCAATTTGGT
Ce (JX976925)               ---------AAAAAGCTGATATTCATCA-AAAAAGGTCAT------GCTGTCAATTTGGT
Hs (Mariner-36_HSal)        AAAGCACTTCTAAAGCAGACATTCACCAAAGGAAGATTTT------GCTATCTGTTTGGT
Hs (Mariner-23_HSal)        AAAGCACATCAAAAGCAGACATCCACCAGAGAAAGGTGAT------GCTCTCAATCTGGT
Pb (PBa_Mariner-23_HSal)    AAAGCACATCAAAAGCAAACATCCACCAGAGGAAAGTGAT------GCTCTCAATCTGAT
Ca (Mariner_CA)             AAACCACTTCAAAGGCTGATATCCACCAAAAGAAGGTTAT------GCTGTCTGTTTGGT
Si (Mariner-24_SIn)         AAAGCACTTCGAAAGCCGATATTCATCAAAAAAAGGTGAT------GCTATCTGTTTGGT
Sm (SMAR7)                  AATCCACTTCGAAGGCCGATATTCACCAAAAAAAGGTTAT------GCTATCTGTTTGGT
Af (Mariner-1_AFl)          AAAGCACTTCGAAAGCCAACATTCACCAAAAAAAAGTGAT------GCTCTCTGTTTGGT
Hs (Mariner-11_HSal)        AAAGCACATCGAAAGCTGATATCCATCAAAAGAAGGTGAT------GCTCTCTGTTTGGT
Hs (Mariner-16_HSal)        AGACGGTCGCAAAGGCTGGATTACACCCGAAGAAGGTCAT------GCTCTGTATCTGGT
```

```
Der (Mariner-2_DEr)           AAACAGTGGCCAAGCCGGGATTGACGGCCAGGAAGGTTTT------GCTGTGTGTTTGGT
Hs (Mariner-26_HSal)          AAACGATCGCCAAGCCCGGATTAACGGCCAAGAAGGTTTT------ACTGTGTGTTTGGT
Del (Mariner-2_ DEl)          AGGCGGTGGCTAAGCCAGGATTGACGGCCAGAAAGGTTTT------GCTGTGTGTTTGGT
Del (Mariner-1_ DEl)          AGACGGTGGCCAAGCCTGGATTGACGGCCAGGAAGGTTCT------TCTGTGTGTTTGGT
Bt (Mariner-1_BT)             CAACCACACCAAAGGCCGGTCTTCATCCAAAGAAGGTGAT------GTTGTGTATATGGT
Ac (Mariner-5_ACe)            TAGCCACCCCAAAAGCCGGTCTTCATCCAAAGAAGGTCAT------GCTCTGTGTCTGGT
Si (Mariner-28_SIn)           TGAC CACTCCAAAGCCTGGGCTTCATCCGGAGAAGATCAT------GCTGTGTATTTGAT
                                          *        *              *           *     * *  *

Hs (Mariner-24_SIn)           GGGATTTCAAGGGTG--TCATCTATTACGAACTGTTACCGTCAGGCAAAACGATTGATTC
Hs (Mariner-45_HSal)          GGGATTGGAAGGGCA--TCGTGCATCACGAGCTGCTGCCGCCAGGCAGAACGATAGACTC
Cf (Mariner-6_CFl)            GGAATTGGAAGGGGA--TAGTATATTATGAGCTACTACCGGACAACGAAACCATCGATTC
Hs (Mariner -35_HSal)         GGGATTGGAAAGGAA--TCGTGTA CTACGAGCTGCTTCCGCAAAACCAAACAATTGATTC
Df (Mariner-1-DF)             GGAATTGGAAGGGTG--TAGTGCACTTTGAGCTGCTGGGACGGAATGAAACCATCAATTC
Ac (Mariner-1_ACe)            GGGATTGGAAGGGAC--CAGTCTTCTATGAGCTACTTCCAAAGAACAAAACGATTAATTC
Lh (ADOQ01008024)             GGGATTGGAAGGGAC--CAGTCTTCTATGAGCTGCTTCCAAAGAATAAAACGATTAATTC
Lh (ADOQ01001582)             GGGATTGGAAGGGAC--CAGTCTTCTATGAGCTGCTTCCAAAGAATAAAACGATTAATTC
Dm (JX976930)                 AGG---------GTG--CTATGTCTTTCGAACTCCTACTAGGGA ACCAAAGAATCAATTC
Dm (JX976937)                 AGG---------GTG--CTATGTCTTTCGAACTCCTACTAGGGAACCAAAGAATCAATTC
Dm (JX976938)                 GGA---------GTG--CCATGTCTTTCGAATTCCCACTAAGGAACCAAAGAATCAATTC
Ce (JX976929)                 GGA---------GTG--CCATGTCTTTCGAATTCCCACTAAGGAACCAAAGAATCAATTC
Dm (JX976934)                 GGA---------GTG--CCATGTCTTTCGAATTCCCACTAAGGAACCAAAGAATCAATTC
Ce (JX976928)                 GGA---------GTG--CCATGTCTTTCGAATTCCCACTAAGGAACCAAAGAATCAATTC
Dm (JX976936)                 GGA---------GTG--CCATGTCTTTCGAATTCCCACTAAGGAACCAAAGAATCAATTC
Mr (AFJA01006902)             GGGATTTCAAAGGCA--CAGTGTTTTCCGAACTCTTATCAAGAAATCAAACGATCAATTC
Mr (AFJA01006736)             GGGATTTGAAAGGCA--TAGTGTTTTTCGAATTCCTATCAAGAAATCAAACGATCAATTC
Dm (JX976932)                 GGGATTTTAAAGGTG--ACATGTTTTTCAAGTTCCTGCCAAGAAACCAAACAATCAATTA
Dm (JX976933)                 GGGATTTTAAAGGTG--ACATGTTTTTCAAGTTCCTGCCAAGAAACCAAACAATCAATTA
Si (AEAQ01010279)             GAGATGTT AAAGGTA--TCGTGTTTCTCGAGCTCCTACCAAGGAACCAAACAATCAATTG
Si (AEAQ01009575)             GGAATTTTAAAGGTG--TCGTGTTTTTCGAGCTCCCACCAAGAAACCAAATAATCAATTC
Cf (AEAB01001421)             GGGATTGGAAGGGTG--TGGTGTTTTTTGAGCCCCTACCAAGGAACCGAACAATCAATTC
Cf (AEAB01018477)             GGGATTGGAAGGGTG--TGGTGTTTTTTGAGCCCCTACCAAGGAACCGAACAATCAATTC
Ee (EEu_Mariner_Tbel)         GGGATTTTAAAGGCA--TT-----TTTTGAGCTCCTACCAAGGAACCAAACAATCAATTC
Pb (PBa_Mariner_Tbel)         GGGATTTTAAAGGTG--TCGTGTTTTTC GAGCTCCTACCAAGGAACCAAACAATCAATTC
Tb (Mariner_Tbel)             GGGATTTTAAAGGTG--TCGTGTTTTTCGAGCTCCTACCAAGGAACCAAACAATCAATTC
Hs (HSal_Mariner_Tbel)        GGGATTTTAAAGGTG--TCGTGTTTTTCGAGCTCCTACCAAGGAACCAAACAATCAATTC
Hs (Mariner-22_HSal)          GGGATTGGAAGGGTG--TTGTGTTCTACGAGCTACTTCCGAAGAACAGAACGATCAATTC
Ae (Mariner-16_AEc)           GGGATTGGAAGGGAA--TCGTTCACTATGAGCTGTTATCGCCCGATCAAACAATTAATTC
Ae (AEc_Mariner-8_Sln)        GGGATTGGAAGGGAG--TCGTCCATCATGAGGTGTTACCACATGGCCT AACGATTAATTC
Si (Mariner-8_SIn)            GGGATTGGAAGGGAG--TCGTCCATTATGAGGTGTTACCACATGGCCTAACGATTAATTC
Bte (Mariner-1_Bte)           GGGATTATAAAGGAA--TTCTGTACTTTGAACTTTTACCAAGAAACCAAACGATTAATTC
Am (AMe_FAMAR1)               GGGATTACAAAGGAA--TTGTCTATTTTGAACTCTTACCACCCAACCGAACGATCAATTC
Fa (FAMAR1)                   GGGATTACAAAGGAA--TTGTCTACTTTGAACTCTTACCACCCAACCGAACGATCAATTC
Hs (Mariner-2_HSal)           GGGATTACAAAGGAA--TTGTCTACTTTGAAATTTTACCACGCAACCAAACGATCAATTC
Hs (Mariner-42_HSal)          GGGATTACAAAGGTG--TTGTGTATTTTGAGATGCTTCCAAGCAACCAAACGATCAACTC
Ac (Mariner-13_ACe)           GGGATTTCAAAGGTG--TTGTGTATTTTGAGCTGCTTCCAAGGAATCAAACGATTAATTC
Ae (AEVX01012963)             GGGATTTCAAAGGTG--TTGTGTATTTTGAGCTGCTTCCAAGGAATCAAACGATTAATTC
Cc_4                          GGGATTACAAAGGTG--TTGTGTATTTCGGACTGTTTCCAAGGAATCAGACC--------
Cc_6                          GGGATTACAAAGGTG--TTGTGTATTTCGGACTGTTTCCAAGGAATCAGACC--------
Cc_5                          GGGATTACAAAG GTG--TTGTGTATTTCGATCGGTTTCCAAGGAATCAGACC--------
Cc_7                          GGGATTACAAGGGTG--TTGTGTATTTCGGACTGTTTCCAAGGAATCAGACC--------
Cc (JX976920)                 GGAATTACAAACGTA--TTGTGTATTTTGAGCTATTTCCCAGGAACCAGACGATCAATTC
Cc (JX976921)                 GGAATTACAAACGTA--TTGTGTATTTTGTGCTATTTCCCAGGAACCAGACGATCAATTC
Cc (JX976922)                 GGAATTACAAACGTA--TTGTGTATTTTGAGCTATTTCCCAGGAACCAGACGATCAATTC
Cc (JX976923)                 GGAATTACAAACGTA--TTGTGTATTTTGAGC TATTTCCCAGGAACCAGACGATCAATTC
Dm (JX976931)                 GGAA--------------------TTTTGAGCTTCTTCCAAGGGAGCAGA--ATAAATTT
Ce (JX976927)                 GGAATTACAAAGGCT--GTGTGCATTTTGAGTTGCCTCCAAGGAACCAGACAATCAATTC
Dm (JX976935)                 GGAATTACAAAGGCT--GTGTGCATTTTGAGTTGCCTCCAAGGAACCAGACAATCAATTC
Ce (JX976926)                 GGAATTACAAAGGCT--GTGTGCATTTTGAGTTGCCTCCAAGGAACCAGACAATCAATTC
Ce (JX976924)                 GGAATTACAAAGGCT--GTGTGCATTTTGAGTTGCCTCCAAGGAACCAGACA ATCAATTC
Ce (JX976925)                 GGAATTACAAAGGCT--GTGTGCATTTTGAGTTGCCTCCAAGGAACCAGACAATCAATTC
Hs (Mariner-36_HSal)          GGGATTACAAGGGTA--TTGTGTTTTTTGAACTGCTTAAACGTAATCAAACGATTGATTC
Hs (Mariner-23_HSal)          GGGATTGGAAGGGTG--TGGTGTTTTTCGAGCTCTTACCAAGGAATGTTACGATCAATTC
Pb (PBa_Mariner-23_HSal)      GGGATTGGAAGGGTG--TGGTGTTTTTCGAGCTCTTACCAAGGAATGTTTCGATCAATTC
Ca (Mariner_CA)               GGGATTGGAAGGGTG--TGGTATATTTTGAGCTGCTTCCAAGGAACCAAACGATTAATTC
```

```
Si (Mariner-24_SIn)   GGGATTTCAAAGAAATCTTTTTTTTTTTGAGCTTCTACCGGACAACACAACGATCAATTC
Sm (SMAR7)            GGGATTTCAAGGGGA--TCGT-TTTTTTGAGCTTCTACCGGACAATACCACGATTAATTC
Af (Mariner-1_AFl)    GGGATTTCAAGGGAG--TTGTTTTTTTTGAGCTTCTACCTGACAATTGCACGATTAATTC
Hs (Mariner-11_HSal)  GGGATTGGAAAGGAA--TGGTTTTTTTTGAGCTGCTACCAAACAACCAAACGATTGATTC
Hs (Mariner-16_HSal)  GGGATTGGAAAGGAA--TCGTCTATTATGAGCTGCTGCCACCCAACAAAACGATTGATTC
Der (Mariner-2_DEr)   GGGATTGGAAGGGAA--TCATCCACTATGAGCTGCTCCCATATGGCCAGACGCTTAATTC
Hs (Mariner-26_HSal)  GGGA TTGGAAGGGAA--TCATCCACTATGAGCTGCTCCCATCGGGCCAATCACTCAATTC
Del (Mariner-2_ DEl)  GGGATTGGCAAGGAA--TTATCTACTATGAGCTGCTCCCCTATGGCCAAACACTTAATTC
Del (Mariner-1_ DEl)  GGGATTGGCAGGGAA--TAATCCACTATGAGCTGCTCCCCTATGGCCAAACGCTCAATTC
Bt (Mariner-1_BT)     GGGATTGGAAGGGAG--TCCTCTATTATGAGCTCCTTCCGGAAAACCAAACGATTAATTC
Ac (Mariner-5_ACe)    GGGATTGGAAAGGGA--TCCTGTATTATGAGCTTTTACCAAACAACGAGACGATAAATTC
Si (Mariner-28_SIn)   GGGACTGGAA-GGTG--TCGTGTA TTATGAGCTCCTTCCAAAGAACGTACGGCTTAATTC
```

```
Hs (Mariner-24_SIn)    AACTGTATACTGTTCGCAATTGACGAAATT----GAACCAAGCAATCCGCACAAAACG--
Hs (Mariner-45_HSal)   GGACCTGTACTGTCGACAATTAGCGCGATT----GCACCTAGCAATTCAAAAGAAACG--
Cf (Mariner-6_CFl)     GGACAAGTACTGTTCACAGTTGGATAAATT----GAAGATAGAAATCGCCAAAAAGTG--
Hs (Mariner -35_HSal)  CAACAAGTATTGTTCCCAATTGGACTGTTT----GAAGGCAGCA ATCGATGAGAAGCG--
Df (Mariner-1-DF)      AGATGTGTACTGTCGCCAGCTATCCAATTT----GGCGGAAAAAATTAAAGAAAAGGA--
Ac (Mariner-1_ACe)     CGATGTCTACTGTGAGCAGCTGCAGAAATT----AAGTGATGCCATCGCACAGAAACG--
Lh (ADOQ01008024)      CGATGTCTCCTGTGAGCAGCTACAGAAATT----AAGTGATGCCATCGCACAGAAACG--
Lh (ADOQ01001582)      CGATGTCTACTGTGAGCAGCTACAGAAATT----AAGTGATGCCATCGCACAGAAACG--
Dm (JX976930)          AAATCCATACTGTTAACAATTT------------GAACGAATCCGTTACCTAGAAAGG--
Dm (JX976937)          AAATCCATACTGTTAACAATTT------------GAACGAATCCGTTACCTAGAAAGG--
Dm (JX976938)          AAATCTATACTGTTAACAATTT------------GAACGAATCTGTTATCAAGAAAGG--
Ce (JX976929)          AAATCTATACTGTTAACAATTT------------GAACGAATCTGTTATCTAGAAAGG--
Dm (JX976934)          AAATCTATACTGTTAACAATTT------------GAACGAATCTGTTATCTAGAAAGG--
Ce (JX976928)          AAATCTATACTGTTAACAATTT------------GAACGAATCTGTTATCTAGAAAGG--
Dm (JX976936)          AAATCTAT ACTGTTAACAATTT------------GAACGAATCTGTTATCTAGAAAGG--
Mr (AFJA01006902)      TGACGTCTACTGCCGGCAATTAGAAAGTTT----GAAAGAATCAATTGAACAAAAACG--
Mr (AFJA01006736)      TGATATCTACTGCCGGTAATTAGAGAGTTT----GAAAGAATCAATTGAACAAAAACG--
Dm (JX976932)          GAGTGTGTACTATCAGCAATTGGACAGTTTAAAAGAAAGAATCCTACGTGCAGAAACA--
Dm (JX976933)          GAGTGTGTACTATCAGCAATTGGACAGTTTAAAAGAAAGAATCCTTCGTGCAGAAACA--
Si (AEAQ01010279)      AAACGTGTACTGTTGGCAATTGGACAGT TT----TAACGAATCTATCACCTAGAAATG--
Si (AEAQ01009575)      GCGTATGTACTGTCGGCAATTGGACAATTT----AAACAAATCCATCATCCAGAAACGTT
Cf (AEAB01001421)      GGATGTGTACTGTCGGCAATTGGACAGTTT----GAACGAATCTGTCATCCAGAAACG--
Cf (AEAB01018477)      GGATGTGTACTGTCGGCAATTGGACAGTTT----GAACGAATCTGTCATCCAGAAACG--
Ee (EEu_Mariner_Tbel)  AAATGTGTACTGTCGGCAATTGGACAGTTT----GAACGAATCCATCATCCAGAAATG--
Pb (PBa_Mariner_Tbel)  GAATGTGTACTGTCGGCAATTGGACAGTTT----GAACGAATCCATCA TCCAGAAACG--
Tb (Mariner_Tbel)      GAATGTGTACTGTCGGCAATTGGACAGTTT----GAACGAATCCATCATCCAGAAACG--
Hs (HSal_Mariner_Tbel) GAATGTGTACTGTCGGCAATTGGACAGTTT----GAACGAATCCATCATCCAGAAACG--
Hs (Mariner-22_HSal)   AGATGTGTACTGTAGTCAGCTGGATAAATT----GAATGCAGCGATCCACGAGAAGCG--
Ae (Mariner-16_AEc)    CGAACTCTACTGTGAACAACTGGAGAGATT----ACAACAAGCAATTGAGAGGAAGCG--
Ae (AEc_Mariner-8_Sln) AGAGCTCTACTGTTCACAACTGGATAGATT----ACAGGAAGCGATTAAGGAAAAACG--
Si (Mariner-8_SIn)     AGAGCTCTACTGTTCACAACTGGATAGATT----ACAGGAAGCGATTAAGGAAAAACG--
Bte (Mariner-1_Bte)    AAACGTGTACGTTCAACAACTCGCCAAACT----GAGCGATGCAGTTCAAGAAAAGCG--
Am (AMe_FAMAR1)        TGTTGTCTACATTGAACAACTAACGAAATT----AAACAATGCAGTTGAAGAAAAGCG--
Fa (FAMAR1)            TGTTGTCTACATTGAACAACTAACGAAATT----AAACAATGCAGTTGAAGAAAAGCG--
Hs (Mariner-2_HSal)    AGATGTATACATTCAACAACTAACCAAACT----GAACAATGCAATTCAAGAAAAGCG--
Hs (Mariner-42_HSal)   AGATGTTTACTG CCAACAACTTATGAAATT----GGAGGAAGCAATCAAAGAGAAAAG--
Ac (Mariner-13_ACe)    AGATGTCTACTGCCAACAACTAATGAAACT----GGAGGAAGCAATCAAAGAAAAACG--
Ae (AEVX01012963)      AGATGTCTACTGCCAACAACTAATGAAACT----GGAGGAAGCAATTAAAGAAAAACG--
Cc_4                   ----------------------------------GGATGAAGCAATCAAAGAAAAGCT--
Cc_6                   ----------------------------------GGATGAAGCAATCAAAGAAAAGCT--
Cc_5                   ----------------------------------GGATGAAGCAATCAAAGAAAAGCT--
Cc_7                   ----------------------------------GGATGAAGCAATCAAAGAAAAGCT--
Cc (JX976920)          AGATCCTTATTGTTAACAACTTATGAAAAT----GGATGAAGCAACCAAAGAAAAGCT--
Cc (JX976921)          AAATCCTTATTGTTAACAACTTATGAAAAT----GGATGAAGCAACCAAAGAAAAATA--
Cc (JX976922)          AAATCCTTATTGTTAACAACTTATGAAAAT----GGATGAAGCAACCAAAGAAAAATA--
Cc (JX976923)          AAATCCTTATTGTTAACAACTTATGAAAAT----GGATGAAGCAACCAAAGA AAAATA--
Dm (JX976931)          ATATGTTTCCTGTTAACAACTAATGAAACT----GGATGAAGTAATCAAAGAAAAACG--
Ce (JX976927)          ACATGTTTACTGTCAACAACTAATAAAACT----GAATGAAGCAATCAAAGAAAAACG--
Dm (JX976935)          ACATGTTTACTGTCAACAACTAATAAAACT----AAATGAAGCAATCAAAGAAAAACG--
Ce (JX976926)          ACATGTTTACTGTCAACAACTAATAAAACT----GAATGAAGCAATCAAAGAAAAACG--
Ce (JX976924)          ACATGTTTACTGTCAACAACTAATAAAACT----GAATGAAGCAATCAAAGAAAAACG--
```

```
Ce (JX976925)                ACATGTTTACTGTCAACAACTAATAAAACT----GAATGAAGCAATCAAAGAAAAACG--
Hs (Mariner-36_HSal)         GGAATTGTACTGTCGTCAATTGGACAAATT----ACACGAAGCAATCAAGCAGAAGCG--
Hs (Mariner-23_HSal)         GGACGTGTACTGTCAACAGCTCGACAAATT----GAACGAAGCAATAGCAGAAAAGCG--
Pb (PBa_Mariner-23_HSal)     GGACGTGTACTGTCAACAGCTAGACAAACT----GAACGAAGCAATAGCAGAAAAGCG--
Ca (Mariner_CA)              GGATGTTTACTGTCAACAACTGGACAAATT----GAATGCAGCCATCAACGAGAAACG--
Si (Mariner-24_SIn)          TGAAGTGTACTGTCATCAGCTGGACAAATT----GAATGATTCACTTAAACAGAAAAG--
Sm (SMAR7)                   TGAAGTGTACTGTGATCAACTGGACAAATT----GAATGATTCGCTCAAACAGAAAAG--
Af (Mariner-1_AFl)           GGAAGTGTACTGCAATCAATTGGACAAATT----AAACGATTCCATCAAACAAAAGAG--
Hs (Mariner-11_HSal)         GAATGTGTATTGTCATCAATTGGACAAATT----GAATGATTCCATCAAGCAGAAGAG--
Hs (Mariner-16_HSal)         GACCAAGTACTGCTCACAACTGGCCAAATT----AAAGCGAGCAATCGATCAGAAGCG--
Der (Mariner-2_DEr)          TACCATCTACTGCGAACAACTGGACCGCTT----GAAGCAGGCGATCGACCAGAAGCG--
Hs (Mariner-26_HSal)         GGACCTACACTGTCAACAACTGACCAGATT----GAAGCAGGCGATCGACGAGAAGCG--
Del (Mariner-2_ DEl)         GGTTTTGTACTGTCAACAATTGGACCGTCT----GAAGGAAGCAATTGCCCAGAAGCG--
Del (Mariner-1_ DEl)         GGACCTGTACTGCCAACAACTGGACCGCTT----GAAGGCAGCACTCATGCAGAAGAG--
Bt (Mariner-1_BT)            CAACAAGTACTGCTCCCAGTTAGACCAACT----GAAAGCAGCACTCGACGAAAAGCG--
Ac (Mariner-5_ACe)           AGAGAAGTATTGTTCCCAATTAGACGAATT----GAAGACAGCAATTGAACAAAAACG--
Si (Mariner-28_SIn)          GGACAAGTACTGTGCCCAATTGGACAAACT----GAAGGAAGCCATTGCAGAAAAACG--
                                                                                **

Hs (Mariner-24_SIn)          ----TCCAGAATTGGCAAAT-CGCAAGGGTGTCGTCTTCCACCACGACAACGCCAGACCT
Hs (Mariner-45_HSal)         ----GCCGGAACTGGTCAAC-AGAAAGGGTGTCGTATACCACGCTGACAACGCCAGACCG
Cf (Mariner-6_CFl)           ----TCCGGAATTGATCAAT-AGGAAAGGCGTCGTTTTTCATCATGATAACGCCAGACCT
Hs (Mariner -35_HSal)        ----TCCAGAATTGAGCAAT-CGTTATGGTGTCATATTCCACCAAGACAACGCTAGACCT
Df (Mariner-1-DF)            ----GCCGGCACTAGCTAAT-CGCAAGGGTATAGTCTTTCACCATGACAACGCTAGGCCC
Ac (Mariner-1_ACe)           ----TCCCGAGCTAATCAAT-CGTAAGGGTGTGGTGTTTCACCACGACAATGCGAGACCA
Lh (ADOQ01008024)            ----TCCCGAGCTAATCAAT-CGTAAGGGTGTGGTGTTTCACCACGACAATGCGAGACCA
Lh (ADOQ01001582)            ----TCCCGAGCTGATCAAT-CGTAAGGGTGTGGTGTTTCACCACGACAATGTGAGACCA
Dm (JX976930)                ----TGCAAAGCACCTTAAT-AAGTTAGGATTGGCATTTCATCGCGGCAATGCAAGGCCA
Dm (JX976937)                ----TGCAAAGCACCTTAAT-AAGTTAGGATTGGCATTTCATCGCGGCAATGCAAGGCCA
Dm (JX976938)                ----TGCAGAGCTTCTTAATAAGTAAAGGAGTGGCGTTTTCTGACGGCAATGCAAGGCCA
Ce (JX976929)                ----TGTAGAGCTTCTTAATAAGTAAAGGAGTGGCGTTTTCTGACGGCAATGCAAGGCCA
Dm (JX976934)                ----TGCAGAGCTTCTTAATAAGTAAAGGAGTGGCGTTTTCTGACGGCAATGCAAGGCCA
Ce (JX976928)                ----TGCAGAGCTTCTTAATAAGTAAAGGAGTGGCGTTTTCTGACGGCAATGCAAGGCCA
Dm (JX976936)                ----TGCAGAGCTTCTTAATAAGTAAAGGAGTGGCGTTTTCTGACGGCAATGCAAGGCCA
Mr (AFJA01006902)            ----CCCAGAACTAGCCAAC-CGTAAAGGAGTTGTGTTCCAACACGATAATGCGAGACCA
Mr (AFJA01006736)            ----CCCAGAACTGGCCAAT-CGTAAAGGATTTATGTTCCACCCCGATAATGCGAGACCA
Dm (JX976932)                ----TCCAGAGCTTGTTAAC-CATAAAGGAGTTGTATTTCATCATGACAATGCAAGGCCA
Dm (JX976933)                ----TCCAGAGCTTGTTAAC-CATAAAGGAGTTGTATTTCATCATGACAATGCAAGGCCA
Si (AEAQ01010279)            ----TATA-AATTCATTAAT-CGTAAAGGAGTTGTGTTCCATCACGACAACGCGTGACCA
Si (AEAQ01009575)            AATATCCAGAACTCGTTAAC-CATAAAGAAGTTGTGTTCCATCACGACAACGCAAGACCA
Cf (AEAB01001421)            ----TCCGGAGCTCGTCAAT-CGTAAAGGAGTTGTGTTCCATCACGACAACGCGAGACCA
Cf (AEAB01018477)            ----TCCGGAGCTCGTCAAT-CGTAAAGGAGTTGTGTTCCATCACGACAACGCGAGACCA
Ee (EEu_Mariner_Tbel)        ----TCCAGAGCTCGTTAAT-CGTAAAGGAGTTGTGTTCCATCATGACAACGCAAGACCA
Pb (PBa_Mariner_Tbel)        ----TCCAGAGCTCGTTAAT-CGTAAAGGAGTTGTGTTCCATCACGACAACGCGAGACCA
Tb (Mariner_Tbel)            ----TCCAGAGCTCGTTGAT-CGTAAAGGAGTTGTGTTCCATCACGACAACGCGAGACCA
Hs (HSal_Mariner_Tbel)       ----TCCAGAGCTCGTTAAT-CGTAAAGGAGTTGTGTTCCATCACGACAACGCGAGACCA
Hs (Mariner-22_HSal)         ----TCCAGAATTGGTGAAT-CGTAGAGGCGTCGTCTTTCACCATGACAACGCTAGGCCG
Ae (Mariner-16_AEc)          ----GCCAGAATTAATCAAT-AGGAGGGGTGTCGTCTTCCATCACGACAACGCTCGACCA
Ae (AEc_Mariner-8_Sln)       ----ACCAGAATTGATTAAC-AGAAAAGGTGTTGTCTTCCATCATGACAACGCCAGACCA
Si (Mariner-8_SIn)           ----ACCAGAATTGATCAAC-AGAAAAGGTGTTGTCTTCCATCATGACAACGCCAGACCA
Bte (Mariner-1_Bte)          ----GCCAGAATTGGCAAAT-CGTAAGGGTGTTGTTTTCCAGCATGATAATGCAAAGCCC
Am (AMe_FAMAR1)              ----GCCCGAATTGACAAAT-CGAAAAGGTGTTGTATTCCATCATGACAATGCAAGGCCA
Fa (FAMAR1)                  ----GGCCGAATTGACAAAT-CGAAAAGGTGTTGTATTCCATCATGACAATGCAAGGCCA
Hs (Mariner-2_HSal)          ----ACCAGAATTGGCAAAT-CGAAAAGGTATTGTGTTCCACCATGACAATGCAAGGCCA
Hs (Mariner-42_HSal)         ----GCCAGAATTGGCAAAT-CGTAAAGGAATCGTGTTCCACCACGACAATGCGAGGCCC
Ac (Mariner-13_ACe)          ----GCCAGAGTTAGCGAAT-CGCAAAGGAATCGTCTTTCATCATGACAATGCAAGACCA
Ae (AEVX01012963)            ----GCCAGAGTTAGCGAAT-TGCAAAGGAATCGTCTTTCATCATGACGATGCAAGACCA
Cc_4                         ----GCCATAATCACCACAT-CACATACGAATTGTGTTCGATCATGATAATGCAAGGCCA
Cc_6                         ----GCCATAATCGGCACAT-CACATACGAATTGTGTTCGATCATGATAATGCAAGGCCA
Cc_5                         ----GCCATAATTGGCAAAT-CACATAGGAACTGTGTTCGATCATGAT----CAAGGCCA
Cc_7                         ----GCCATAATTGGCAAAT-CACATAGGAACTGTGTTCGATCATGAT----CAAGGCCA
Cc (JX976920)                ----GCCATAATTGGCAAAT-CACATAGGAACTGTGTTCGATCATGAT----CAAGGCCA
Cc (JX976921)                ----GCTAAAATTGGAAAAT-CGCTATAGAATTATGTTCCACCATGTTAAAGCAAGGCCA
Cc (JX976922)                ----GCTAAAATTGGAAAAT-CGCTATAGAATTGTGTTCCACCATGTTAAAGCAAGGCCA
Cc (JX9723)                  ----GCTAAAATTGGAAAAT-CGCTATAGAATTGTGTTCCACCATGTTAAAGCAAGGCCA
```

```
Dm (JX976931)                 ----GCCAAATTTGGCAAAT-CGCAAGGGAGTTGTGTTCCACCGTGATAATGCAAGGCCA
Ce (JX976927)                 ----ATCAGAATTGGCAAAT-CGCAAAGGAATTGTGTTCCACCATGATAATGCAAGGCCA
Dm (JX976935)                 ----ATCAGAATTGGCAAAT-CGCAAAGGAATTGTGTTCCACCATGATAATGCAAGGCCA
Ce (JX976926)                 ----ATCAGAATTGGCAAAT-CGCAAAGGAATTGTGTTCCACCATGATAATGCAAGGCCA
Ce (JX976924)                 ----ATCAGAATTGGCAAAT-CGCAAAGGAATTGTGTTCCACCATGATAATGCAAGGCCA
Ce (JX976925)                 ----ATCAGAATTGGCAAAT-CGCAAAGGAATTGTGTTCCACCATGATAATGCAAGGCCA
Hs (Mariner-36_HSal)          ----CCCAGAACTGGTGAAT-CGTAAAGATGTTGTCTTTCACCATGACAACGCTAGACCA
Hs (Mariner-23_HSal)          ----GCCAGAATTGATAAAT-CGCAAAGGAGTGGTCTTCCATCATGACAACGCTCGACCA
Pb (PBa_Mariner-23_HSal)      ----GCCAGAATTGATAAAT-CGCAAAGGAGTAGTATTCCAACATGACAACGCTCGACCA
Ca (Mariner_CA)               ----GCCAGAATTGATCAAT-CGTAAAGGTGTCATATTCCATCAGGACAACGCCAGACCA
Si (Mariner-24_SIn)           ----GCCAGAATTGATCAAT-AGAAAAGGTGTAGTGTTCCACCAAGATAATGCGAGACCT
Sm (SMAR7)                    ----GCCAGAATTGATCAAT-AGAAAAGGTATAGTGTTCCACCACGATAATGCGAGACCT
Af (Mariner-1_AFl)            ----ATCTGAATTAATTAAC-AGGAAAGATGTAGTGTTTCATCACGATAACGCTAGACCT
Hs (Mariner-11_HSal)          ----ACCAGAATTGGCGAAC-AGAAAAGGTGTTGTGTTTCATCATGACAACGCCAGACCT
Hs (Mariner-16_HSal)          ----GCCCGAATTGGCGAAC-AGAAAGGGCGTTGTGTTCCACCAGGACAACGCTAGACCA
Der (Mariner-2_DEr)           ----TCCAGAATTGGCCAAC-AGGAAGGGTGTAGTGTTCCACCAGGACAACGCCAGACCA
Hs (Mariner-26_HSal)          ----GCCAGAATTGGCCAAT-AGGAAGGGTGTTGTGTTCCATCAAGACAATGCCAGGCCG
Del (Mariner-2_ DEl)          ----CCCCGCTTTGGCCAAT-AGGAAAGGAATTGTTTTCCATCAGGACAACGCTAGACCA
Del (Mariner-1_ DEl)          ----GCCATCTTTGATCAAC-AGAGGCCGAATTGTCTTCCATCAGGACAACGCCAGGCCA
Bt (Mariner-1_BT)             ----TCCGGAATTAGTCAAC-AGAAAACGCATAATCTTCCATCAGGATAACGCAAGACCG
Ac (Mariner-5_ACe)            ----TCCAGAAATAGCGAAT-CGGAAGGGCGTCGTGTTTCATCAGGACAATGCGCGGCCT
Si (Mariner-28_SIn)           ----CCCAGAATTGGTGAAT-AGAAAGGGTGTTTTATTCCATCAGGACAACGCCAGGCCT
                                                *                 *        *           **

Hs (Mariner-24_SIn)           CATACGTCATTGACCACTCGGAA-
Hs (Mariner-45_HSal)          CATACATCTTTAACGACCCGTCAG
Cf (Mariner-6_CFl)            CATGCAAGTTTGCAGACTCGCCAA
Hs (Mariner -35_HSal)         CACGTATCTTTGGCAACCCGGCAA
Df (Mariner-1-DF)             CACACATCTTTGGTCACTCGGCA-
Ac (Mariner-1_ACe)            CACACAAGTTTGGTCACTCGGC--
Lh (ADOQ01008024)             CACACAAGTTTGGTCACTCGGC--
Lh (ADOQ01001582)             CACACAAGTTTGGTCACTCGGC--
Dm (JX976930)                 CACACATCTTTGA-----------
Dm (JX976937)                 CACACATCTTTGA-----------
Dm (JX976938)                 CACACATCTTTGA-----------
Ce (JX976929)                 CACACATCTTTGA-----------
Dm (JX976934)                 CACACATCTTTGA-----------
Ce (JX976928)                 CACACATCTTTGA-----------
Dm (JX976936)                 CACACATCTTTGA-----------
Mr (AFJA01006902)             CACACAAGTTTGGTCACCCGC---
Mr (AFJA01006736)             CAGACGAGTGTGGTCACCCGC---
Dm (JX976932)                 CACACATCTTTGA-----------
Dm (JX976933)                 CACACATCTTTGA-----------
Si (AEAQ01010279)             TACATAAGTTTGATCAC-------
Si (AEAQ01009575)             CACACAAATTTGATCAG-------
Cf (AEAB01001421)             CACACATCTTTGGC----------
Cf (AEAB01018477)             CACACATCTTTGGC----------
Ee (EEu_Mariner_Tbel)         CACACAAGTTTGATCACCTGT---
Pb (PBa_Mariner_Tbel)         CACACAAGTTTGACCACCCGT---
Tb (Mariner_Tbel)             CACACAAGTTTGATCACCCGT---
Hs (HSal_Mariner_Tbel)        CACACAAGTTTGATCACCCGT---
Hs (Mariner-22_HSal)          CACACATCTCTACAAACCCGGCAA
Ae (Mariner-16_AEc)           CACACATCTTTGATAACTCGGCAA
Ae (AEc_Mariner-8_Sln)        CACACATCTTTGATGACGCGCCAA
Si (Mariner-8_SIn)            CACACATCTTTGATGACGCGCCAA
Bte (Mariner-1_Bte)           CACACATCTTTGGTCACTCGCCA-
Am (AMe_FAMAR1)               CACACATCTTTGGTCACTCGGCA-
Fa (FAMAR1)                   CACACATCTTTGGTCACTCGGCA-
Hs (Mariner-2_HSal)           CACACATCTTTGGTCACTCGGCA-
Hs (Mariner-42_HSal)          CATACATCTTTAGCAACACGTAC-
Ac (Mariner-13_ACe)           TACACATCTTTGGCCACGCGGCA-
Ae (AEVX01012963)             TACACATCTTTGGCCACGCGGCA-
Cc_4                          CACACATCTTTGTTGA--------
Cc_6                          CACACATCTTTGGA----------
Cc_5                          CACACATCTTTGTTGA--------
```

```
Cc_7                        CACACATCTTTGTTGA--------
Cc (JX976920)               CACACATCTTTGGA----------
Cc (JX976921)               CACACATCTTTGTTTG--------
Cc (JX976922)               CACACATCTTTGGA----------
Cc (JX976923)               CACACATCTTTGTTTGA-------
Dm (JX976931)               CACACATCTTTGCTTTG-------
Ce (JX976927)               CACACATCTTTGGA----------
Dm (JX976935)               CACACATCTTTGGA----------
Ce (JX976926)               CACACATCTTTGTTTGA-------
Ce (JX976924)               CACACATCTTTGGA----------
Ce (JX976925)               CACACATCTTTGTTTGA-------
Hs (Mariner-36_HSal)        CATACATCGTTGGTCACCCGCCA-
Hs (Mariner-23_HSal)        CACACAAGTTTGATGACTCGCG--
Pb (PBa_Mariner-23_HSal)    CATACAAGTTTGATGACTCGCA--
Ca (Mariner_CA)             CACACATCTTTGATGACCCGGCA-
Si (Mariner-24_SIn)         CATACAAGTTTGGTAACTCGTCA-
Sm (SMAR7)                  CATACAAGTTTGGTAACTCGCCA-
Af (Mariner-1_AFl)          CACACGAGTTTAATGACTCGCCA-
Hs (Mariner-11_HSal)        CACACAAGTTTAATGACTCGCCA-
Hs (Mariner-16_HSal)        CACGTTTCTCTGATGACCCGGCAA
Der (Mariner-2_DEr)         CACACTTCGTTGATGACTCGTCAG
Hs (Mariner-26_HSal)        CACACATCTTTGACGACGCGCCAG
Del (Mariner-2_ DEl)        CACACGTCAATAGCGACTCGCCAG
Del (Mariner-1_ DEl)        CACACATCTTTAGTGACGCGCCAG
Bt (Mariner-1_BT)           CATGTTTCTTTGATGACCAGGCAA
Ac (Mariner-5_ACe)          CATGTGTCTTTGATAACTCGACA-
Si (Mariner-28_SIn)         CATGTTTCTTTAACCACCCGGAA-
                             *        *
```
