# Supplementary material for: Chromosomal organization and evolutionary history of Mariner transposable elements in Scarabaeinae coleopterans
Source: Mol Cytogenet. 2013 Nov 29;6:54. doi: 10.1186/1755-8166-6-54 (PMC3906913; doi:10.1186/1755-8166-6-54)

Additional File 3: Figure S2 - Alignment guide tree of *Mariner* families based on Maximum Likehood. The taxa are the same as described in Figure 3. The bootstrap support values are indicated on the nodes. The scale bar indicates the genetic distance.

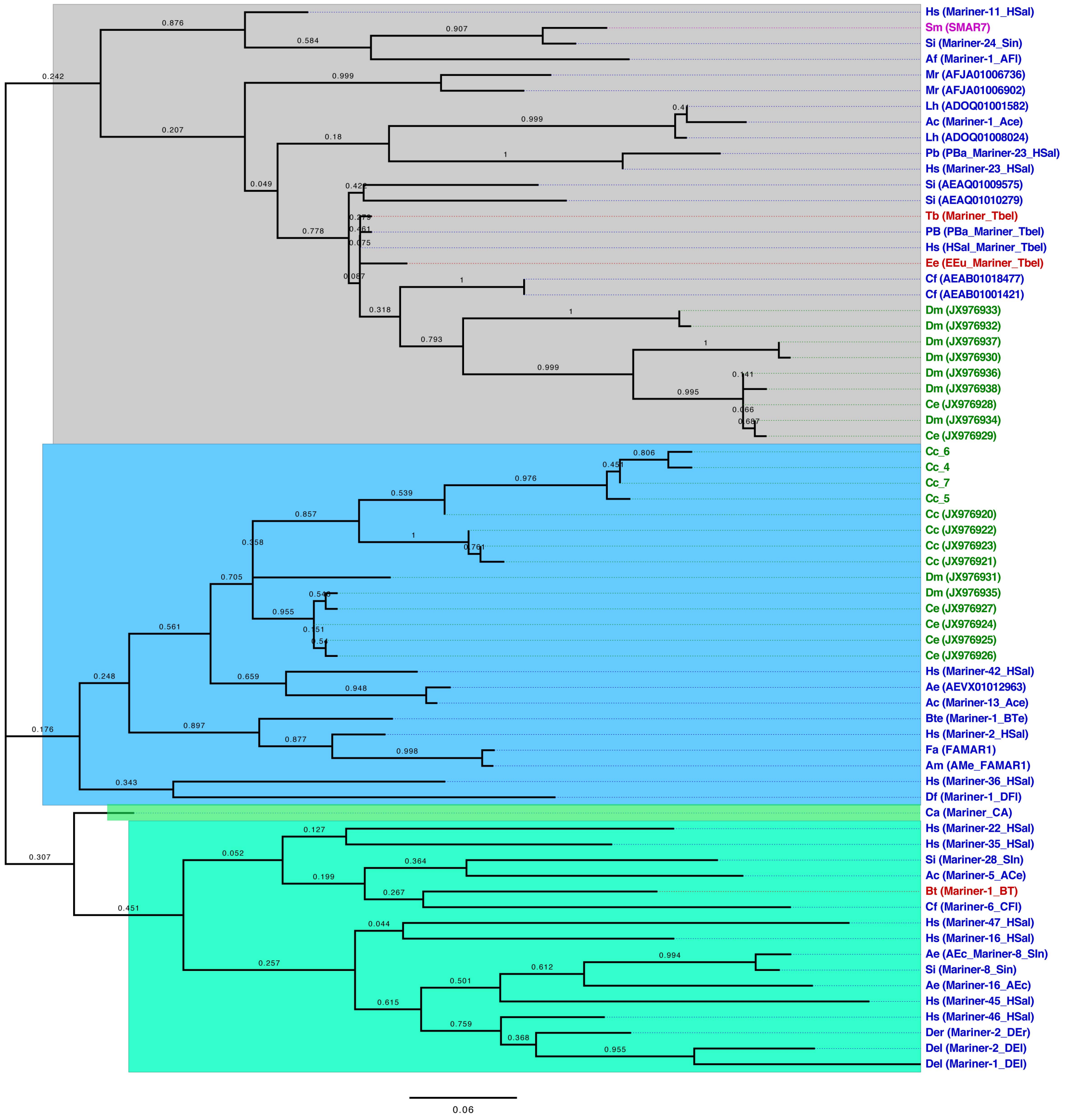

Supplement: Additional file 3: Figure S2 — Alignment guide tree of Mariner families based on Maximum Likehood. The taxa are the same as described in Figure 3. The bootstrap support values are indicated on the nodes. The scale bar indicates the genetic distance. [file 1755-8166-6-54-S3.pdf]
