# Supplementary material for: Chromosomal organization and evolutionary history of Mariner transposable elements in Scarabaeinae coleopterans
Source: Mol Cytogenet. 2013 Nov 29;6:54. doi: 10.1186/1755-8166-6-54 (PMC3906913; doi:10.1186/1755-8166-6-54)
Supplement: Additional file 4: Dataset S2 — Alignments pairwise similarity matrix of related Mariner families. The abbreviations correspond to the species names and IDs, as shown in Figure 3. [file 1755-8166-6-54-S4.pdf]

Additional File 4: Dataset S2 - Pairwise similarity alignment matrix of related *Mariner* families. The abbreviations correspond to the species names and IDs as described in the caption of Figure 2.

Sequences	Hs (Mariner-47_HSal)	Hs (Mariner-45_HSal)	Cf (Mariner-6_CFl)	Hs (Mariner-35_HSal)	Df (Mariner-1_DF)	Ac (Mariner-1_ACe)	Lh (ADOQ01001582)	Lh (ADOQ01008024)	Dm (JX976930)
**Hs (Mariner-47_HSal)**	ID								
**Hs (Mariner-45_HSal)**	0.665	ID							
**Cf (Mariner-6_CFl)**	0.685	0.632	ID						
**Hs (Mariner-35_HSal)**	0.669	0.653	0.722	ID					
**Df (Mariner-1_DF)**	0.639	0.628	0.644	0.653	ID				
**Ac (Mariner-1_ACe)**	0.647	0.583	0.657	0.653	0.676	ID			
**Lh (ADOQ01001582)**	0.635	0.583	0.640	0.648	0.684	0.971	ID		
**Lh (ADOQ01008024)**	0.635	0.579	0.644	0.644	0.676	0.930	0.950	ID	
**Dm (JX976930)**	0.520	0.461	0.489	0.469	0.528	0.539	0.526	0.527	ID
**Dm (JX976937)**	0.516	0.461	0.485	0.473	0.532	0.543	0.530	0.531	0.995
**Dm (JX976938)**	0.514	0.455	0.483	0.467	0.518	0.536	0.524	0.533	0.844
**Ce (JX976929)**	0.514	0.451	0.483	0.463	0.514	0.532	0.520	0.529	0.853
**Dm (JX976934)**	0.526	0.467	0.495	0.475	0.534	0.549	0.536	0.537	0.882
**Ce (JX976928)**	0.522	0.467	0.487	0.475	0.538	0.553	0.540	0.541	0.877
**Dm (JX976936)**	0.514	0.455	0.483	0.471	0.522	0.540	0.528	0.537	0.853
**Mr (AFJA01006902)**	0.643	0.604	0.616	0.636	0.663	0.707	0.703	0.679	0.557
**Mr (AFJA01006736)**	0.635	0.608	0.616	0.640	0.655	0.699	0.691	0.674	0.549
**Dm (JX976932)**	0.572	0.518	0.534	0.558	0.576	0.607	0.591	0.600	0.659
**Dm (JX976933)**	0.584	0.534	0.546	0.570	0.596	0.623	0.607	0.609	0.685
**Si (AEAQ01010279)**	0.608	0.529	0.601	0.629	0.596	0.626	0.614	0.617	0.622
**Si (AEAQ01009575)**	0.604	0.545	0.593	0.605	0.604	0.630	0.618	0.621	0.605
**Cf (AEAB01001421)**	0.647	0.612	0.636	0.624	0.668	0.687	0.670	0.683	0.668
**Cf (AEAB01018477)**	0.647	0.612	0.636	0.624	0.668	0.687	0.670	0.683	0.668
**Ee (EEu_Mariner_Tbel)**	0.655	0.587	0.653	0.648	0.672	0.703	0.691	0.674	0.648
**Pb (PBa_Mariner_Tbel)**	0.688	0.624	0.653	0.685	0.684	0.728	0.716	0.699	0.665
**Tb (Mariner_Tbel)**	0.688	0.608	0.657	0.677	0.684	0.728	0.716	0.699	0.661
**Hs (HSal_Mariner_Tbel)**	0.692	0.612	0.661	0.681	0.688	0.732	0.720	0.703	0.665
**Hs (Mariner-22_HSal)**	0.693	0.644	0.734	0.718	0.693	0.710	0.689	0.689	0.530
**Ae (Mariner-16_AEc)**	0.677	0.706	0.657	0.689	0.644	0.673	0.673	0.665	0.489
**Ae (AEc_Mariner-8_SIn)**	0.689	0.710	0.685	0.669	0.640	0.653	0.648	0.640	0.497
**Si (Mariner-8_Sin)**	0.685	0.710	0.689	0.685	0.648	0.665	0.661	0.653	0.497
**Bte (Mariner-1_Bte)**	0.668	0.604	0.616	0.657	0.700	0.684	0.672	0.663	0.549
**Am (AMe_FAMAR1)**	0.688	0.608	0.640	0.624	0.692	0.700	0.696	0.688	0.569
**Fa (FAMAR1)**	0.680	0.600	0.632	0.624	0.692	0.700	0.696	0.688	0.569
**Hs (Mariner-2_HSal)**	0.704	0.595	0.624	0.644	0.737	0.684	0.680	0.672	0.565
**Hs (Mariner-42_HSal)**	0.692	0.628	0.632	0.657	0.696	0.680	0.676	0.663	0.565
**Ac (Mariner-13_ACe)**	0.704	0.608	0.644	0.657	0.745	0.713	0.717	0.700	0.569
**Ae (AEVX01012963)**	0.692	0.604	0.636	0.644	0.737	0.700	0.704	0.688	0.573
**Cc_4**	0.479	0.408	0.457	0.473	0.520	0.473	0.481	0.481	0.515
**Cc_6**	0.495	0.420	0.465	0.489	0.536	0.477	0.485	0.497	0.524
**Cc_5**	0.479	0.412	0.457	0.473	0.520	0.465	0.473	0.481	0.497
**Cc_7**	0.487	0.416	0.461	0.469	0.524	0.469	0.477	0.485	0.502
**Cc (JX976920)**	0.577	0.493	0.567	0.555	0.622	0.576	0.563	0.570	0.590
**Cc (JX976921)**	0.573	0.493	0.546	0.559	0.602	0.567	0.555	0.569	0.588
**Cc (JX976922)**	0.598	0.506	0.571	0.575	0.627	0.584	0.572	0.579	0.607
**Cc (JX976923)**	0.581	0.502	0.555	0.559	0.610	0.576	0.563	0.577	0.594
**Dm (JX976931)**	0.549	0.489	0.518	0.493	0.586	0.576	0.584	0.589	0.589
**Ce (JX976927)**	0.598	0.538	0.575	0.587	0.659	0.633	0.633	0.634	0.588
**Dm (JX976935)**	0.598	0.534	0.575	0.587	0.659	0.637	0.637	0.650	0.590
**Ce (JX976926)**	0.594	0.538	0.571	0.579	0.655	0.629	0.629	0.644	0.590
**Ce (JX976924)**	0.602	0.546	0.587	0.600	0.668	0.646	0.646	0.640	0.590
**Ce (JX976925)**	0.590	0.542	0.575	0.583	0.659	0.633	0.633	0.648	0.590
**Hs (Mariner-36_HSal)**	0.668	0.640	0.657	0.673	0.713	0.684	0.688	0.684	0.536
**Hs (Mariner-23_HSal)**	0.655	0.604	0.718	0.657	0.717	0.724	0.736	0.728	0.563
**Pb (PBa_Mariner23_HSal)**	0.651	0.608	0.693	0.648	0.709	0.699	0.716	0.707	0.543
**Ca (Mariner_CA)**	0.700	0.677	0.710	0.738	0.737	0.762	0.754	0.754	0.581
**Si (Mariner-24_SIn)**	0.638	0.607	0.700	0.655	0.662	0.715	0.707	0.703	0.524
**Sm (SMAR7)**	0.655	0.620	0.706	0.657	0.672	0.729	0.725	0.729	0.516
**Af (Mariner-1_AFl)**	0.631	0.575	0.693	0.624	0.647	0.688	0.692	0.680	0.512
**Hs (Mariner-11_HSal)**	0.672	0.628	0.718	0.697	0.684	0.725	0.721	0.713	0.553
**Hs (Mariner-16_HSal)**	0.697	0.697	0.714	0.734	0.628	0.673	0.669	0.661	0.469
**Der (Mariner-2_DEr)**	0.685	0.718	0.636	0.673	0.608	0.657	0.648	0.636	0.465
**Hs (Mariner-46_HSal)**	0.677	0.722	0.657	0.669	0.640	0.640	0.632	0.624	0.493
**Del (Mariner-2_DEl)**	0.624	0.685	0.644	0.665	0.608	0.608	0.600	0.591	0.489
**Del (Mariner-1_DEl)**	0.575	0.697	0.620	0.628	0.604	0.595	0.587	0.575	0.477
**Bt (Mariner-1_BT)**	0.653	0.636	0.730	0.738	0.636	0.636	0.636	0.628	0.497
**Ac (Mariner-5_ACe)**	0.676	0.624	0.722	0.702	0.663	0.643	0.643	0.647	0.520
**Si (Mariner-28_SIn)**	0.663	0.648	0.697	0.714	0.631	0.639	0.622	0.631	0.514

Sequences	Dm (JX976937)	Dm (JX976938)	Ce (JX976929)	Dm (JX976934)	Ce (JX976928)	Dm (JX976936)	Mr (AFJA01006902)	Mr (AFJA01006736)	Dm (JX976932)	Dm (JX976933)	Si (AEAQ01010279)
**Hs (Mariner-47_HSal)**											
**Hs (Mariner-45_HSal)**											
**Cf (Mariner-6_CFl)**											
**Hs (Mariner-35_HSal)**											
**Df (Mariner-1_DF)**											
**Ac (Mariner-1_ACe)**											
**Lh (ADOQ01001582)**											
**Lh (ADOQ01008024)**											
**Dm (JX976930)**											
**Dm (JX976937)**	ID										
**Dm (JX976938)**	0.849	ID									
**Ce (JX976929)**	0.849	0.980	ID								
**Dm (JX976934)**	0.877	0.962	0.971	ID							
**Ce (JX976928)**	0.882	0.962	0.962	0.990	ID						
**Dm (JX976936)**	0.858	0.990	0.990	0.971	0.971	ID					
**Mr (AFJA01006902)**	0.561	0.559	0.555	0.572	0.576	0.563	ID				
**Mr (AFJA01006736)**	0.553	0.547	0.543	0.559	0.563	0.551	0.909	ID			
**Dm (JX976932)**	0.655	0.679	0.684	0.673	0.669	0.684	0.654	0.634	ID		
**Dm (JX976933)**	0.681	0.669	0.673	0.699	0.695	0.673	0.670	0.650	0.974	ID	
**Si (AEAQ01010279)**	0.626	0.619	0.628	0.640	0.640	0.628	0.705	0.673	0.673	0.693	ID
**Si (AEAQ01009575)**	0.609	0.615	0.611	0.632	0.636	0.619	0.697	0.681	0.722	0.742	0.773
**Cf (AEAB01001421)**	0.672	0.673	0.669	0.686	0.690	0.678	0.735	0.714	0.754	0.771	0.784
**Cf (AEAB01018477)**	0.672	0.673	0.669	0.686	0.690	0.678	0.735	0.714	0.754	0.771	0.784
**Ee (EEu_Mariner_Tbel)**	0.652	0.641	0.637	0.654	0.658	0.646	0.776	0.760	0.739	0.756	0.806
**Pb (PBa_Mariner_Tbel)**	0.669	0.662	0.658	0.679	0.683	0.666	0.801	0.785	0.768	0.788	0.842
**Tb (Mariner_Tbel)**	0.665	0.662	0.658	0.674	0.679	0.666	0.809	0.793	0.768	0.784	0.834
**Hs (HSal_Mariner_Tbel)**	0.669	0.666	0.662	0.679	0.683	0.670	0.814	0.797	0.772	0.788	0.838
**Hs (Mariner-22_HSal)**	0.526	0.524	0.528	0.540	0.536	0.528	0.648	0.640	0.598	0.610	0.625
**Ae (Mariner-16_AEc)**	0.485	0.471	0.471	0.487	0.483	0.471	0.632	0.612	0.538	0.554	0.561
**Ae (AEc_Mariner-8_Sln)**	0.493	0.491	0.495	0.512	0.512	0.495	0.632	0.616	0.554	0.570	0.593
**Si (Mariner-8_Sin)**	0.502	0.500	0.495	0.512	0.520	0.504	0.644	0.628	0.554	0.570	0.589
**Bte (Mariner-1_Bte)**	0.553	0.551	0.546	0.563	0.563	0.555	0.713	0.680	0.608	0.625	0.632
**Am (AMe_FAMAR1)**	0.565	0.538	0.542	0.563	0.559	0.542	0.692	0.676	0.608	0.629	0.604
**Fa (FAMAR1)**	0.565	0.538	0.542	0.563	0.559	0.542	0.684	0.668	0.600	0.620	0.600
**Hs (Mariner-2_HSal)**	0.569	0.555	0.551	0.567	0.571	0.559	0.700	0.692	0.604	0.620	0.616
**Hs (Mariner-42_HSal)**	0.569	0.571	0.567	0.587	0.587	0.575	0.688	0.692	0.604	0.625	0.624
**Ac (Mariner-13_ACe)**	0.573	0.567	0.563	0.579	0.579	0.571	0.709	0.696	0.616	0.633	0.632
**Ae (AEVX01012963)**	0.577	0.571	0.567	0.583	0.583	0.575	0.704	0.692	0.608	0.625	0.620
**Cc_4**	0.511	0.500	0.504	0.504	0.495	0.504	0.524	0.500	0.536	0.536	0.497
**Cc_6**	0.529	0.495	0.491	0.513	0.513	0.500	0.541	0.524	0.531	0.553	0.510
**Cc_5**	0.493	0.486	0.490	0.491	0.482	0.490	0.516	0.500	0.536	0.536	0.497
**Cc_7**	0.497	0.490	0.495	0.495	0.486	0.495	0.516	0.500	0.532	0.531	0.493
**Cc (JX976920)**	0.594	0.566	0.562	0.583	0.583	0.570	0.603	0.590	0.597	0.614	0.597
**Cc (JX976921)**	0.584	0.578	0.592	0.581	0.573	0.583	0.578	0.578	0.610	0.604	0.597
**Cc (JX976922)**	0.603	0.575	0.587	0.600	0.592	0.579	0.599	0.590	0.605	0.622	0.618
**Cc (JX976923)**	0.590	0.585	0.598	0.587	0.579	0.589	0.586	0.578	0.616	0.610	0.605
**Dm (JX976931)**	0.584	0.597	0.601	0.600	0.595	0.601	0.557	0.561	0.590	0.588	0.551
**Ce (JX976927)**	0.593	0.635	0.631	0.629	0.629	0.640	0.652	0.644	0.662	0.659	0.613
**Dm (JX976935)**	0.594	0.622	0.618	0.630	0.630	0.626	0.648	0.640	0.648	0.661	0.630
**Ce (JX976926)**	0.594	0.637	0.633	0.630	0.630	0.641	0.648	0.640	0.663	0.661	0.626
**Ce (JX976924)**	0.594	0.637	0.633	0.630	0.630	0.641	0.657	0.657	0.668	0.665	0.610
**Ce (JX976925)**	0.594	0.634	0.630	0.630	0.630	0.639	0.644	0.644	0.665	0.665	0.622
**Hs (Mariner-36_HSal)**	0.540	0.530	0.526	0.538	0.542	0.534	0.725	0.713	0.612	0.625	0.648
**Hs (Mariner-23_HSal)**	0.567	0.561	0.557	0.577	0.577	0.565	0.736	0.716	0.623	0.643	0.702
**Pb (PBa_Mariner23_HSal)**	0.547	0.528	0.524	0.545	0.549	0.532	0.724	0.703	0.603	0.623	0.686
**Ca (Mariner_CA)**	0.586	0.571	0.567	0.583	0.587	0.575	0.692	0.696	0.653	0.669	0.668
**Si (Mariner-24_Sln)**	0.528	0.522	0.518	0.530	0.530	0.526	0.699	0.695	0.588	0.600	0.634
**Sm (SMAR7)**	0.520	0.518	0.514	0.526	0.526	0.522	0.704	0.700	0.580	0.592	0.644
**Af (Mariner-1_AFl)**	0.516	0.502	0.497	0.510	0.510	0.506	0.676	0.655	0.572	0.584	0.644
**Hs (Mariner-11_HSal)**	0.557	0.546	0.542	0.559	0.563	0.551	0.709	0.700	0.657	0.673	0.664
**Hs (Mariner-16_HSal)**	0.465	0.459	0.463	0.479	0.475	0.463	0.624	0.620	0.566	0.582	0.577
**Der (Mariner-2_DEr)**	0.461	0.455	0.459	0.471	0.467	0.459	0.612	0.608	0.538	0.550	0.537
**Hs (Mariner-46_HSal)**	0.489	0.483	0.487	0.500	0.495	0.487	0.604	0.595	0.554	0.566	0.545
**Del (Mariner-2_DEl)**	0.485	0.467	0.471	0.483	0.475	0.471	0.604	0.620	0.546	0.558	0.545
**Del (Mariner-1_DEl)**	0.473	0.451	0.455	0.467	0.463	0.455	0.583	0.571	0.534	0.546	0.521
**Bt (Mariner-1_BT)**	0.493	0.475	0.479	0.495	0.491	0.479	0.608	0.608	0.558	0.574	0.561
**Ac (Mariner-5_ACe)**	0.516	0.510	0.514	0.526	0.522	0.514	0.651	0.647	0.584	0.596	0.596
**Si (Mariner-28_Sln)**	0.510	0.495	0.500	0.512	0.508	0.500	0.610	0.606	0.568	0.580	0.584

Sequences	Si (AEAQ01009575)	Cf (AEAB01001421)	Cf (AEAB01018477)	Ee (EEu_Mariner_Tbel)	Pb (PBa_Mariner_Tbel)	Tb (Mariner_Tbel)	Hs (HSal_Mariner_Tbel)	Hs (Mariner-22_HSal)	Ae (Mariner-16_AEc)
**Hs (Mariner-47_HSal)**									
**Hs (Mariner-45_HSal)**									
**Cf (Mariner-6_CFl)**									
**Hs (Mariner-35_HSal)**									
**Df (Mariner-1_DF)**									
**Ac (Mariner-1_ACe)**									
**Lh (ADOQ01001582)**									
**Lh (ADOQ01008024)**									
**Dm (JX976930)**									
**Dm (JX976937)**									
**Dm (JX976938)**									
**Ce (JX976929)**									
**Dm (JX976934)**									
**Ce (JX976928)**									
**Dm (JX976936)**									
**Mr (AFJA01006902)**									
**Mr (AFJA01006736)**									
**Dm (JX976932)**									
**Dm (JX976933)**									
**Si (AEAQ01010279)**									
**Si (AEAQ01009575)**	ID								
**Cf (AEAB01001421)**	0.784	ID							
**Cf (AEAB01018477)**	0.784	1,000	ID						
**Ee (EEu_Mariner_Tbel)**	0.794	0.822	0.822	ID					
**Pb (PBa_Mariner_Tbel)**	0.846	0.871	0.871	0.925	ID				
**Tb (Mariner_Tbel)**	0.838	0.863	0.863	0.933	0.983	ID			
**Hs (HSal_Mariner_Tbel)**	0.842	0.867	0.867	0.938	0.987	0.995	ID		
**Hs (Mariner-22_HSal)**	0.621	0.710	0.710	0.689	0.714	0.718	0.722	ID	
**Ae (Mariner-16_AEc)**	0.557	0.616	0.616	0.608	0.624	0.624	0.628	0.648	ID
**Ae (AEc_Mariner-8_SIn)**	0.585	0.632	0.632	0.640	0.653	0.653	0.657	0.677	0.787
**Si (Mariner-8_Sin)**	0.589	0.640	0.640	0.644	0.657	0.657	0.661	0.677	0.795
**Bte (Mariner-1_Bte)**	0.608	0.655	0.655	0.696	0.692	0.692	0.696	0.677	0.636
**Am (AMe_FAMAR1)**	0.608	0.663	0.663	0.680	0.680	0.680	0.684	0.661	0.697
**Fa (FAMAR1)**	0.600	0.655	0.655	0.672	0.672	0.672	0.676	0.661	0.697
**Hs (Mariner-2_HSal)**	0.620	0.659	0.659	0.704	0.696	0.700	0.704	0.677	0.661
**Hs (Mariner-42_HSal)**	0.608	0.688	0.688	0.688	0.713	0.713	0.717	0.718	0.628
**Ac (Mariner-13_ACe)**	0.616	0.696	0.696	0.709	0.721	0.721	0.725	0.722	0.648
**Ae (AEVX01012963)**	0.604	0.684	0.684	0.696	0.709	0.709	0.713	0.710	0.653
**Cc_4**	0.495	0.525	0.525	0.508	0.524	0.528	0.528	0.510	0.465
**Cc_6**	0.506	0.560	0.560	0.524	0.545	0.545	0.545	0.526	0.465
**Cc_5**	0.493	0.534	0.534	0.512	0.528	0.528	0.533	0.514	0.457
**Cc_7**	0.489	0.534	0.534	0.508	0.524	0.524	0.528	0.518	0.461
**Cc (JX976920)**	0.585	0.655	0.655	0.628	0.623	0.619	0.623	0.620	0.546
**Cc (JX976921)**	0.556	0.615	0.615	0.615	0.603	0.603	0.607	0.595	0.530
**Cc (JX976922)**	0.576	0.650	0.650	0.640	0.628	0.623	0.628	0.620	0.542
**Cc (JX976923)**	0.564	0.621	0.621	0.623	0.611	0.611	0.615	0.604	0.538
**Dm (JX976931)**	0.551	0.595	0.595	0.616	0.586	0.586	0.590	0.571	0.514
**Ce (JX976927)**	0.621	0.679	0.679	0.673	0.665	0.661	0.665	0.644	0.559
**Dm (JX976935)**	0.643	0.693	0.693	0.677	0.665	0.661	0.665	0.640	0.563
**Ce (JX976926)**	0.634	0.676	0.676	0.673	0.661	0.661	0.665	0.636	0.559
**Ce (JX976924)**	0.627	0.676	0.676	0.685	0.677	0.673	0.677	0.648	0.567
**Ce (JX976925)**	0.639	0.672	0.672	0.677	0.665	0.665	0.669	0.632	0.563
**Hs (Mariner-36_HSal)**	0.668	0.721	0.721	0.733	0.745	0.750	0.754	0.734	0.657
**Hs (Mariner-23_HSal)**	0.682	0.728	0.728	0.740	0.777	0.777	0.781	0.738	0.669
**Pb (PBa_Mariner23_HSal)**	0.682	0.703	0.703	0.716	0.761	0.753	0.757	0.710	0.640
**Ca (Mariner_CA)**	0.672	0.754	0.754	0.745	0.774	0.774	0.778	0.771	0.714
**Si (Mariner-24_SIn)**	0.603	0.670	0.670	0.715	0.711	0.719	0.723	0.692	0.647
**Sm (SMAR7)**	0.612	0.680	0.680	0.695	0.709	0.717	0.721	0.702	0.661
**Af (Mariner-1_AFl)**	0.648	0.655	0.655	0.692	0.713	0.713	0.717	0.677	0.640
**Hs (Mariner-11_HSal)**	0.668	0.704	0.704	0.766	0.766	0.774	0.778	0.710	0.665
**Hs (Mariner-16_HSal)**	0.601	0.612	0.612	0.628	0.648	0.648	0.653	0.689	0.718
**Der (Mariner-2_DEr)**	0.553	0.600	0.600	0.620	0.624	0.628	0.632	0.632	0.746
**Hs (Mariner-46_HSal)**	0.549	0.620	0.620	0.624	0.640	0.636	0.640	0.648	0.755
**Del (Mariner-2_DEl)**	0.553	0.632	0.632	0.624	0.636	0.632	0.636	0.624	0.697
**Del (Mariner-1_DEl)**	0.525	0.600	0.600	0.600	0.600	0.604	0.608	0.624	0.702
**Bt (Mariner-1_BT)**	0.577	0.620	0.620	0.620	0.632	0.632	0.636	0.689	0.657
**Ac (Mariner-5_ACe)**	0.592	0.639	0.639	0.643	0.659	0.659	0.663	0.677	0.673
**Si (Mariner-28_SIn)**	0.596	0.651	0.651	0.618	0.663	0.655	0.659	0.706	0.628

Sequences	Ae (AEc_Mariner-8_SIn)	Si (Mariner-8_SIn)	Bte (Mariner-1_BTe)	Am (AMe_FAMAR1)	Fa (FAMAR1)	Hs (Mariner-2_HSal)	Hs (Mariner-42_HSal)	Ac (Mariner-13_ACe)	Ae (AEVX01012963)	Cc_4	Cc_6
Hs (Mariner-47_HSal)											
Hs (Mariner-45_HSal)											
Cf (Mariner-6_CFl)											
Hs (Mariner-35_HSal)											
Df (Mariner-1_DF)											
Ac (Mariner-1_ACe)											
Lh (ADOQ01001582)											
Lh (ADOQ01008024)											
Dm (JX976930)											
Dm (JX976937)											
Dm (JX976938)											
Ce (JX976929)											
Dm (JX976934)											
Ce (JX976928)											
Dm (JX976936)											
Mr (AFJA01006902)											
Mr (AFJA01006736)											
Dm (JX976932)											
Dm (JX976933)											
Si (AEAQ01010279)											
Si (AEAQ01009575)											
Cf (AEAB01001421)											
Cf (AEAB01018477)											
Ee (EEu_Mariner_Tbel)											
Pb (PBa_Mariner_Tbel)											
Tb (Mariner_Tbel)											
Hs (HSal_Mariner_Tbel)											
Hs (Mariner-22_HSal)											
Ae (Mariner-16_AEc)											
Ae (AEc_Mariner-8_SIn)	ID										
Si (Mariner-8_Sin)	0.975	ID									
Bte (Mariner-1_Bte)	0.657	0.665	ID								
Am (AMe_FAMAR1)	0.697	0.702	0.807	ID							
Fa (FAMAR1)	0.689	0.693	0.807	0.991	ID						
Hs (Mariner-2_HSal)	0.681	0.693	0.844	0.901	0.901	ID					
Hs (Mariner-42_HSal)	0.653	0.657	0.733	0.729	0.721	0.770	ID				
Ac (Mariner-13_ACe)	0.685	0.689	0.745	0.750	0.741	0.774	0.856	ID			
Ae (AEVX01012963)	0.689	0.693	0.745	0.750	0.741	0.774	0.848	0.983	ID		
Cc_4	0.473	0.477	0.580	0.577	0.569	0.581	0.598	0.614	0.606	ID	
Cc_6	0.473	0.485	0.600	0.590	0.581	0.606	0.627	0.631	0.622	0.927	ID
Cc_5	0.473	0.477	0.580	0.573	0.565	0.581	0.602	0.610	0.606	0.925	0.886
Cc_7	0.477	0.481	0.580	0.573	0.565	0.581	0.598	0.606	0.602	0.936	0.896
Cc (JX976920)	0.571	0.583	0.691	0.696	0.688	0.717	0.733	0.733	0.729	0.673	0.714
Cc (JX976921)	0.559	0.563	0.670	0.659	0.651	0.680	0.713	0.692	0.684	0.663	0.646
Cc (JX976922)	0.575	0.579	0.691	0.688	0.680	0.709	0.737	0.725	0.717	0.639	0.670
Cc (JX976923)	0.567	0.571	0.679	0.668	0.659	0.688	0.721	0.700	0.692	0.665	0.648
Dm (JX976931)	0.538	0.542	0.631	0.618	0.610	0.639	0.655	0.668	0.659	0.587	0.576
Ce (JX976927)	0.604	0.616	0.720	0.696	0.692	0.750	0.778	0.770	0.762	0.666	0.681
Dm (JX976935)	0.608	0.620	0.724	0.709	0.704	0.754	0.774	0.774	0.766	0.652	0.683
Ce (JX976926)	0.604	0.616	0.720	0.692	0.688	0.745	0.770	0.762	0.754	0.682	0.673
Ce (JX976924)	0.616	0.628	0.724	0.709	0.704	0.762	0.782	0.774	0.766	0.663	0.678
Ce (JX976925)	0.608	0.620	0.716	0.696	0.692	0.750	0.766	0.758	0.750	0.675	0.669
Hs (Mariner-36_HSal)	0.681	0.689	0.717	0.717	0.709	0.729	0.704	0.733	0.721	0.520	0.545
Hs (Mariner-23_HSal)	0.689	0.697	0.729	0.721	0.713	0.713	0.704	0.737	0.729	0.559	0.572
Pb (PBa_Mariner23_HSal)	0.657	0.665	0.704	0.704	0.696	0.713	0.688	0.713	0.704	0.530	0.543
Ca (Mariner_CA)	0.763	0.779	0.737	0.750	0.741	0.750	0.766	0.782	0.774	0.553	0.565
Si (Mariner-24_SIn)	0.659	0.676	0.715	0.735	0.727	0.743	0.727	0.699	0.695	0.487	0.508
Sm (SMAR7)	0.677	0.693	0.704	0.717	0.709	0.725	0.717	0.704	0.692	0.491	0.512
Af (Mariner-1_AFl)	0.673	0.677	0.663	0.676	0.672	0.680	0.655	0.684	0.672	0.471	0.475
Hs (Mariner-11_HSal)	0.718	0.730	0.721	0.725	0.717	0.750	0.717	0.721	0.717	0.512	0.528
Hs (Mariner-16_HSal)	0.718	0.714	0.665	0.685	0.677	0.665	0.665	0.653	0.648	0.481	0.485
Der (Mariner-2_DEr)	0.751	0.751	0.628	0.640	0.640	0.640	0.624	0.616	0.608	0.436	0.436
Hs (Mariner-46_HSal)	0.755	0.755	0.669	0.677	0.677	0.685	0.657	0.653	0.640	0.461	0.473
Del (Mariner-2_DEl)	0.697	0.697	0.636	0.624	0.624	0.628	0.628	0.636	0.636	0.440	0.448
Del (Mariner-1_DEl)	0.738	0.738	0.608	0.600	0.600	0.608	0.628	0.608	0.604	0.440	0.440
Bt (Mariner-1_BT)	0.693	0.702	0.644	0.648	0.640	0.648	0.616	0.673	0.661	0.506	0.497
Ac (Mariner-5_ACe)	0.665	0.669	0.680	0.684	0.676	0.692	0.663	0.680	0.672	0.504	0.504
Si (Mariner-28_SIn)	0.693	0.693	0.663	0.643	0.635	0.631	0.659	0.672	0.676	0.483	0.491

Sequences	Cc_5	Cc_7	Cc (JX976920)	Cc (JX976921)	Cc (JX976923)	Cc (JX976924)	Dm (JX976931)	Ce (JX976927)	Dm (JX976935)	Ce (JX976926)	Ce (JX976924)	Ce (JX976925)	Hs (Mariner-36_HSal)
**Hs (Mariner-47_HSal)**													
**Hs (Mariner-45_HSal)**													
**Cf (Mariner-6_CFl)**													
**Hs (Mariner-35_HSal)**													
**Df (Mariner-1_DF)**													
**Ac (Mariner-1_ACe)**													
**Lh (ADOQ01001582)**													
**Lh (ADOQ01008024)**													
**Dm (JX976930)**													
**Dm (JX976937)**													
**Dm (JX976938)**													
**Ce (JX976929)**													
**Dm (JX976934)**													
**Ce (JX976928)**													
**Dm (JX976936)**													
**Mr (AFJA01006902)**													
**Mr (AFJA01006736)**													
**Dm (JX976932)**													
**Dm (JX976933)**													
**Si (AEAQ01010279)**													
**Si (AEAQ01009575)**													
**Cf (AEAB01001421)**													
**Cf (AEAB01018477)**													
**Ee (EEu_Mariner_Tbel)**													
**Pb (PBa_Mariner_Tbel)**													
**Tb (Mariner_Tbel)**													
**Hs (HSal_Mariner_Tbel)**													
**Hs (Mariner-22_HSal)**													
**Ae (Mariner-16_AEc)**													
**Ae (AEc_Mariner-8_SIn)**													
**Si (Mariner-8_Sin)**													
**Bte (Mariner-1_Bte)**													
**Am (AMe_FAMAR1)**													
**Fa (FAMAR1)**													
**Hs (Mariner-2_HSal)**													
**Hs (Mariner-42_HSal)**													
**Ac (Mariner-13_ACe)**													
**Ae (AEVX01012963)**													
**Cc_4**													
**Cc_6**													
**Cc_5**	ID												
**Cc_7**	0.978	ID											
**Cc (JX976920)**	0.716	0.711	ID										
**Cc (JX976921)**	0.663	0.663	0.854	ID									
**Cc (JX976922)**	0.643	0.639	0.909	0.944	ID								
**Cc (JX976923)**	0.669	0.665	0.858	0.986	0.948	ID							
**Dm (JX976931)**	0.592	0.592	0.693	0.736	0.714	0.745	ID						
**Ce (JX976927)**	0.671	0.666	0.793	0.798	0.798	0.803	0.765	ID					
**Dm (JX976935)**	0.656	0.652	0.822	0.781	0.826	0.786	0.748	0.952	ID				
**Ce (JX976926)**	0.687	0.682	0.786	0.823	0.790	0.837	0.785	0.965	0.944	ID			
**Ce (JX976924)**	0.668	0.663	0.790	0.794	0.794	0.800	0.770	0.986	0.948	0.960	ID		
**Ce (JX976925)**	0.679	0.675	0.782	0.815	0.786	0.828	0.786	0.956	0.944	0.991	0.961	ID	
**Hs (Mariner-36_HSal)**	0.524	0.532	0.622	0.598	0.618	0.606	0.553	0.643	0.647	0.639	0.647	0.635	ID
**Hs (Mariner-23_HSal)**	0.563	0.567	0.654	0.621	0.641	0.629	0.604	0.670	0.670	0.670	0.683	0.674	0.733
**Pb (PBa_Mariner23_HSal)**	0.534	0.539	0.625	0.596	0.617	0.604	0.592	0.654	0.654	0.654	0.666	0.658	0.717
**Ca (Mariner_CA)**	0.557	0.561	0.668	0.655	0.663	0.655	0.627	0.704	0.700	0.704	0.717	0.709	0.778
**Si (Mariner-24_SIn)**	0.487	0.483	0.617	0.605	0.626	0.613	0.573	0.650	0.646	0.646	0.662	0.650	0.727
**Sm (SMAR7)**	0.491	0.495	0.614	0.602	0.622	0.610	0.572	0.643	0.639	0.639	0.651	0.643	0.741
**Af (Mariner-1_AFl)**	0.467	0.471	0.561	0.536	0.553	0.545	0.516	0.594	0.598	0.594	0.606	0.598	0.741
**Hs (Mariner-11_HSal)**	0.520	0.516	0.631	0.606	0.627	0.614	0.577	0.655	0.655	0.655	0.668	0.659	0.770
**Hs (Mariner-16_HSal)**	0.485	0.481	0.559	0.563	0.571	0.571	0.546	0.591	0.595	0.595	0.604	0.600	0.673
**Der (Mariner-2_DEr)**	0.440	0.444	0.510	0.510	0.522	0.518	0.510	0.559	0.555	0.559	0.571	0.563	0.640
**Hs (Mariner-46_HSal)**	0.461	0.465	0.555	0.538	0.551	0.546	0.538	0.604	0.595	0.600	0.616	0.604	0.628
**Del (Mariner-2_DEl)**	0.444	0.440	0.530	0.506	0.522	0.514	0.493	0.567	0.563	0.563	0.575	0.567	0.648
**Del (Mariner-1_DEl)**	0.432	0.436	0.526	0.518	0.530	0.526	0.489	0.538	0.534	0.538	0.551	0.542	0.616
**Bt (Mariner-1_BT)**	0.493	0.497	0.555	0.555	0.563	0.555	0.522	0.583	0.587	0.587	0.595	0.591	0.628
**Ac (Mariner-5_ACe)**	0.495	0.491	0.602	0.594	0.606	0.602	0.573	0.610	0.610	0.614	0.618	0.618	0.647
**Si (Mariner-28_SIn)**	0.487	0.487	0.565	0.561	0.573	0.565	0.543	0.598	0.602	0.602	0.602	0.598	0.655

Sequences	Hs (Mariner-23_HSal)	Pb (PBa_Mariner23_HSal)	Ca (Mariner_CA)	Si (Mariner-24_Sln)	Sm (SMAR7)	Af (Mariner-1_Afl)	Hs (Mariner-11_HSal)	Hs (Mariner-16_HSal)	Der (Mariner-2_DEr)
Hs (Mariner-47_HSal)									
Hs (Mariner-45_HSal)									
Cf (Mariner-6_CFl)									
Hs (Mariner-35_HSal)									
Df (Mariner-1_DF)									
Ac (Mariner-1_ACe)									
Lh (ADOQ01001582)									
Lh (ADOQ01008024)									
Dm (JX976930)									
Dm (JX976937)									
Dm (JX976938)									
Ce (JX976929)									
Dm (JX976934)									
Ce (JX976928)									
Dm (JX976936)									
Mr (AFJA01006902)									
Mr (AFJA01006736)									
Dm (JX976932)									
Dm (JX976933)									
Si (AEAQ01010279)									
Si (AEAQ01009575)									
Cf (AEAB01001421)									
Cf (AEAB01018477)									
Ee (EEu_Mariner_Tbel)									
Pb (PBa_Mariner_Tbel)									
Tb (Mariner_Tbel)									
Hs (HSal_Mariner_Tbel)									
Hs (Mariner-22_HSal)									
Ae (Mariner-16_AEc)									
Ae (AEc_Mariner-8_Sln)									
Si (Mariner-8_Sln)									
Bte (Mariner-1_Bte)									
Am (AMe_FAMAR1)									
Fa (FAMAR1)									
Hs (Mariner-2_HSal)									
Hs (Mariner-42_HSal)									
Ac (Mariner-13_ACe)									
Ae (AEVX01012963)									
Cc_4									
Cc_6									
Cc_5									
Cc_7									
Cc (JX976920)									
Cc (JX976921)									
Cc (JX976922)									
Cc (JX976923)									
Dm (JX976931)									
Ce (JX976927)									
Dm (JX976935)									
Ce (JX976926)									
Ce (JX976924)									
Ce (JX976925)									
Hs (Mariner-36_HSal)									
Hs (Mariner-23_HSal)	ID								
Pb (PBa_Mariner23_HSal)	0.950	ID							
Ca (Mariner_CA)	0.786	0.766	ID						
Si (Mariner-24_Sln)	0.723	0.707	0.747	ID					
Sm (SMAR7)	0.717	0.700	0.774	0.906	ID				
Af (Mariner-1_AFl)	0.737	0.725	0.729	0.804	0.823	ID			
Hs (Mariner-11_HSal)	0.754	0.729	0.811	0.808	0.790	0.823	ID		
Hs (Mariner-16_HSal)	0.661	0.640	0.726	0.635	0.648	0.640	0.722	ID	
Der (Mariner-2_DEr)	0.608	0.591	0.706	0.623	0.653	0.616	0.681	0.755	ID
Hs (Mariner-46_HSal)	0.628	0.612	0.726	0.631	0.644	0.595	0.677	0.726	0.840
Del (Mariner-2_DEl)	0.612	0.587	0.669	0.603	0.608	0.595	0.657	0.693	0.804
Del (Mariner-1_DEl)	0.604	0.575	0.689	0.611	0.624	0.583	0.657	0.685	0.804
Bt (Mariner-1_BT)	0.653	0.648	0.730	0.663	0.669	0.657	0.677	0.742	0.677
Ac (Mariner-5_ACe)	0.672	0.659	0.696	0.686	0.684	0.655	0.709	0.726	0.648
Si (Mariner-28_Sln)	0.672	0.684	0.741	0.630	0.643	0.622	0.680	0.722	0.644

Sequences	Hs (Mariner-46_HSal)	Del (Mariner-2_DEl)	Del (Mariner-1_Del)	Bt (Mariner-1_BT)	Ac (Mariner-5_ACe)	Si (Mariner-28_SIn)
Hs (Mariner-47_HSal)						
Hs (Mariner-45_HSal)						
Cf (Mariner-6_CFl)						
Hs (Mariner-35_HSal)						
Df (Mariner-1_DF)						
Ac (Mariner-1_ACe)						
Lh (ADOQ01001582)						
Lh (ADOQ01008024)						
Dm (JX976930)						
Dm (JX976937)						
Dm (JX976938)						
Ce (JX976929)						
Dm (JX976934)						
Ce (JX976928)						
Dm (JX976936)						
Mr (AFJA01006902)						
Mr (AFJA01006736)						
Dm (JX976932)						
Dm (JX976933)						
Si (AEAQ01010279)						
Si (AEAQ01009575)						
Cf (AEAB01001421)						
Cf (AEAB01018477)						
Ee (EEu_Mariner_Tbel)						
Pb (PBa_Mariner_Tbel)						
Tb (Mariner_Tbel)						
Hs (HSal_Mariner_Tbel)						
Hs (Mariner-22_HSal)						
Ae (Mariner-16_AEc)						
Ae (AEc_Mariner-8_SIn)						
Si (Mariner-8_Sin)						
Bte (Mariner-1_Bte)						
Am (AMe_FAMAR1)						
Fa (FAMAR1)						
Hs (Mariner-2_HSal)						
Hs (Mariner-42_HSal)						
Ac (Mariner-13_ACe)						
Ae (AEVX01012963)						
Cc_4						
Cc_6						
Cc_5						
Cc_7						
Cc (JX976920)						
Cc (JX976921)						
Cc (JX976922)						
Cc (JX976923)						
Dm (JX976931)						
Ce (JX976927)						
Dm (JX976935)						
Ce (JX976926)						
Ce (JX976924)						
Ce (JX976925)						
Hs (Mariner-36_HSal)						
Hs (Mariner-23_HSal)						
Pb (PBa_Mariner23_HSal)						
Ca (Mariner_CA)						
Si (Mariner-24_SIn)						
Sm (SMAR7)						
Af (Mariner-1_AFl)						
Hs (Mariner-11_HSal)						
Hs (Mariner-16_HSal)						
Der (Mariner-2_DEr)						
Hs (Mariner-46_HSal)	ID					
Del (Mariner-2_DEl)	0.775	ID				
Del (Mariner-1_DEl)	0.787	0.820	ID			
Bt (Mariner-1_BT)	0.636	0.628	0.644	ID		
Ac (Mariner-5_ACe)	0.644	0.616	0.575	0.742	ID	
Si (Mariner-28_SIn)	0.665	0.644	0.616	0.742	0.745	ID
